# Engineering Peptide Modulators for T‑Cell Migration by Structural Scaffold Matching

**DOI:** 10.1021/acs.jmedchem.5c00677

**Published:** 2025-08-12

**Authors:** Jasmin Gattringer, Simon Hasinger, Agnes Weidmann, Katarzyna Walczewska-Szewc, Kirtikumar B. Jadhav, Tobias Zrzavy, Anja Steinmaurer, Paulien Baeten, Monika Perisic, Wilson Cochrane, Markus Muttenthaler, Bieke Broux, Dagmar Gotthardt, K. Johan Rosengren, Christian W. Gruber, Roland Hellinger

**Affiliations:** † Center for Physiology and Pharmacology, 31285 Medical University of Vienna, 1090 Vienna, Austria; ‡ Institute of Physics, Faculty of Physics, Astronomy and Informatics, 49577 Nicolaus Copernicus University in Toruń, 87-100 Toruń, Poland; § Faculty of Chemistry, Institute of Biological Chemistry, 27258 University of Vienna, 1090 Vienna, Austria; ∥ Comprehensive Center for Clinical Neurosciences and Mental Health, Medical University of Vienna, 1090 Vienna, Austria; ⊥ Department of Neurology, Medical University of Vienna, 1090 Vienna, Austria; # Department of Immunology and Infection Biomedical Research Institute, 54496 Hasselt University, 3590 Diepenbeek, Belgium; ∇ Vienna Doctoral School in Chemistry, University of Vienna, 1090 Vienna, Austria; ○ School of Biomedical Sciences, 1974 The University of Queensland, Brisbane, Queensland 4072, Australia; ◆ Institute for Molecular Bioscience, The University of Queensland, Brisbane, Queensland 4072, Australia; ¶ Department for Biological Sciences and Pathobiology, Pharmacology and Toxicology, 27260 University of Veterinary Medicine Vienna, 1210 Vienna, Austria

## Abstract

Lymphocyte migration plays a crucial role in the progression of autoimmune and inflammatory diseases, and the inhibition of autoreactive immune cells is an attractive therapeutic strategy. Pepitem is an endogenous modulator of lymphocyte migration. In this study, we implemented a structural scaffold matching approach to engineer of stabilized pepitem-based probes. Prioritizing the native helix–loop–helix structure of pepitem, protein structure databases were mined to identify the structurally closest peptide scaffold. Leveraging this strategy, we developed VhTI-pep 2, inhibiting CD3^+^ T-lymphocyte migration in vitro with a comparable potency (EC_50_ = 10.6 ± 16.5 nM) to pepitem (EC_50_ = 6.0 ± 6.4 nM). Its potency was further extended to T-cell subsets derived from multiple sclerosis patients and highly disease-driving memory and Th1 cell populations. Our approach will guide the design of stabilized peptide probes and future therapeutics, overcoming the challenges associated with flexible and linear peptides.

## Graphical abstract

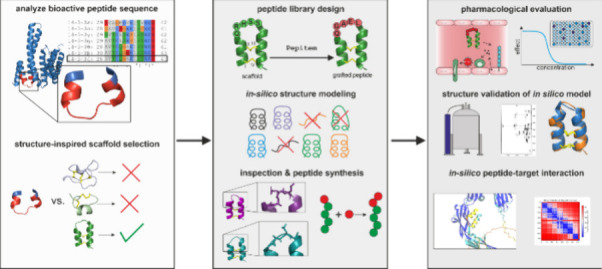

## 1 Introduction

Autoimmune disorders are estimated to impact 5–10% of the population.^1,2^ This group of diseases includes over 100 distinct pathologies, which are characterized by autoreactive immune cells causing inflammation and destruction of body tissues.^3,4^ Immune cell migration throughout the body is a detrimental contribution to the entire immune system. However, aberrant immune cell trafficking can contribute to the development and progression of autoimmune diseases.^3,5^ In multiple sclerosis (MS), peripherally activated immune cells traverse the blood-brain barrier and invade the central nervous system. The following neuroinflammatory processes lead to demyelination and ultimately neurodegeneration.^6,7^ Consequently, obstructing the migration of autoreactive immune cells to prevent central nervous system damage is a promising concept for disease-modifying therapy in MS.^6,8^ The monoclonal anti-integrin antibody, Natalizumab, was the first clinically used immune cell migration modulator for treating MS approved by the FDA in 2004, which was later temporarily withdrawn from the market due to severe adverse events. Today, Natalizumab is in clinical use for relapsing-remitting multiple sclerosis (RRMS) or Crohn’s disease for patients who do not respond to other disease-modifying therapies.^9^ Fingolimod (FTY-720) was the first orally available disease-modifying drug for RRMS which was introduced for clinical use in 2010.^10^ The drug restricts the circulation of T-lymphocytes out of the lymph nodes into the peripheral blood, resulting in a reduction of neuroinflammation and a delay of disease progression.^10–12^ Fingolimod’s major target system is the five sphingosine-1-phosphate receptors (S1PR1–5) expressed on most lymphocytes and also other cells in general, but among others, high expression is reported on pathogenic memory T-cells of the Th1 and Th17 lineages.^13–15^ Today, S1PR subtype-specific drugs (e.g., siponimod, ozanimod, etc.) have been developed for MS as well as for treating ulcerative colitis,^11,16^ and more candidates are in (pre)­clinical development, among others for rheumatoid arthritis or Crohn’s disease.^17,18^ However, S1PR modulators often have severe side effects (e.g., lymphopenia or bradycardia), which limit their use in some patients.^19,20^ In addition, even though the relapse rate in MS patients is reduced with fingolimod, the disease progression is only slowed over time,^19,21^ underscoring the necessity for continued pharmaceutical development in this domain.

The peptide inhibitor of transendothelial migration (pepitem) is a potent endogenous modulator of lymphocyte migration.^22^ Pepitem is enzymatically released from 14-3-3ζ proteins and secreted by adiponectin-stimulated B cells. This signaling molecule binds to the adhesion protein cadherin-15 (CDH15), constitutively expressed on endothelial cells (EC), and activates an effector cascade resulting in endothelial in remodulation and release of the soluble lipid mediator sphingosine-1-phosphate (S1P) into the local microenvironment. The S1PR system is a major regulator of transendothelial lymphocyte migration. Interestingly, the pepitem pathway is dysregulated in autoimmune disorders; e.g., in patients with type 1 diabetes, serum levels of pepitem are reduced in comparison to healthy controls.^22^ Several studies presented that therapeutic dosing of pepitem affects immune cell migration to the site of inflammation and highlight therapeutic benefits of the peptide in autoimmune (e.g., in models of glomerulonephritis or autoimmune uveitis) as well as inflammatory (e.g., obesity-induced inflammation or peritonitis) disease models. The studies mainly focused on T-cells in detail, but effects on B-cell or monocyte migration were evident as well.^22–25^

Therefore, pepitem can be used to study the mechanism of transendothelial immune cell migration and to develop new immune cell migration modulators. Further structural information is necessary to ascertain and define the active conformation of this peptide, thereby facilitating the design of novel pharmacological probes. However, little structure–activity relationship (SAR) knowledge of pepitem exists. For example, a sequence-activity relationship analysis of pepitem did not yield conclusive results regarding its pharmacophore.^26^ Structural investigations were hampered by the flexibility of the linear peptide.^27^ Stabilizing pepitem into its active state would enable further investigations.

Molecular grafting is a prominent tool for stabilizing peptides by embedding the epitopes of interest into a peptide scaffold, resulting in chimeric molecules.^28^ This strategy is also often used in peptide drug development since it can result in rigid bioactive peptide grafts that provide protection against enzymatic degradation in biological fluids.^28–30^ The high momentum of molecular grafting in drug development is reflected by manifold studies using this approach.^31,32^ The selection of an appropriate scaffold peptide is crucial for maintaining bioactivity. Until now, molecular grafting has been used to enhance the stability of linear and flexible peptide sequences,^31^ and common scaffold peptides include sunflower trypsin inhibitor-1 (SFTI-1), kalata B1, and the *Momordica cochinchinensis* trypsin inhibitor-II (MCoTI-II), among others.^28^ However, little attention has been given to the epitope structure during scaffold selection. Because the secondary structure of a peptide greatly affects the orientation of the pharmacophore side chains, not considering the original structural motifs often leads to low success rates in creating bioactive grafts, rendering this a tedious and expensive undertaking. Hence, a more rational method is needed for scaffold selection, where the 3D structure of the original peptide that embeds the bioactive epitope/pharmacophore of interest is considered in the process. The available tools to evaluate the designed chimeric grafts in silico were not powerful enough in the past, reflecting a limitation of this ligand-based grafting approach. An accurate structural prediction and in silico evaluation of multiple grafting designs would considerably reduce costs for synthesis and pharmacological evaluation, and is likely to yield higher success rates to deliver potent and stable chimeric peptide grafts. The recent advancements in artificial intelligence (AI) for modeling and protein or peptide structure predictions have made several breakthroughs in this space, now enabling such studies.^33,34^ However, even though deep learning algorithms provide helpful prediction models, a pharmacological and structural evaluation of the synthesized peptides is needed to confirm the success of the grafting approach.

To implement a structure-guided grafting workflow that reduces the tedious ligand-based design and synthesis efforts, we developed an innovative approach, termed structural scaffold matching, which combines structure-based scaffold mining and in silico predictions for engineering stabilized peptides. Here, we employed this approach to generate new stabilized and bioactive probes of pepitem. Sequence alignment analysis of the endogenous pepitem amino acid sequence provided initial starting structural information. Using structure-based scaffold searches, we identified helix–loop–helix natural peptide scaffolds suitable for incorporating pepitem. Utilizing the combinatorial workflow, we designed and selected grafted variants with a high conformational fit for the embedded bioactive sequence using in silico models. Six representative peptides were biologically characterized. The conformationally stabilized probes provided new opportunities for studies on the molecular mechanism and supported detailed analysis of peptide–protein interaction on the molecular level using AlphaFold (AF) predictions of local interaction sites. In general, this study supports the development of peptide therapeutics that specifically target immune cell migration in the context of autoimmune disorders.

## 2 Results

### 2.1 Pepitem Inhibits T-Cell Migration in a Transwell In Vitro Assay

To investigate the effect of pepitem or other modulators of transendothelial migration (TEM) of immune cells in vitro, we set up a transwell-based migration assay. The general workflow of the assay is illustrated in Figure 1A. First, the endothelial cell (EC) monolayer model (HMEC-1 cells) was characterized for the expression of key proteins for lymphocyte TEM (Figure S1A–C). The conditions for the transwell TEM assay were optimized by measuring the transendothelial transepithelial resistance (TEER) of the monolayer and the permeability of Evans Blue labeled serum albumin through the EC layer (Figure S1D–F). The migration assay was validated using the known inhibitors of lymphocyte migration, S1P (Figure 1B) and lifitegrast (Figure S1G). The reference peptide pepitem was synthesized according to the previously published amino acid sequence (Table 1, Figure S2A) and the bioactivity of pepitem was measured using the transwell migration assay, obtaining an EC_50_ of 6.0 ± 6.4 nM for CD3^+^ lymphocytes (Figure 1C). Previous studies utilized a microscope-based assay that did not resolve or distinguish between T cells, NK cells, and B cells, reporting efficacy in the picomolar range for peripheral blood lymphocytes.^22^ We further evaluated the effects on the migration of CD4^+^ and CD8^+^ T cell populations as well as their memory phenotypes (CD4^+^ or CD8^+^ and CD45RO^+^) (Table 1, Figure S3), where a low nanomolar EC_50_ for the inhibition of migration was established as well.

**Figure 1:**
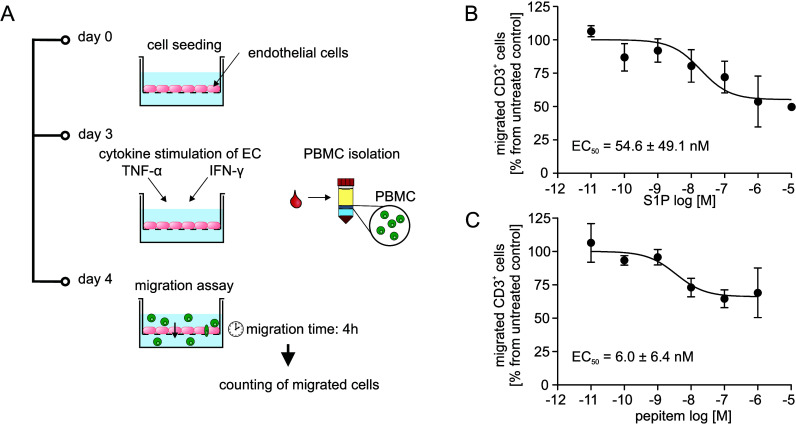
**Transwell-based migration assay to study lymphocyte transendothelial migration**. (A) The general principle of the transwell migration assay is depicted. Endothelial cells (EC) are seeded in transwell inserts and are incubated for 2 days to form an EC monolayer. On the day before the assay, the ECs are cytokine-stimulated to induce the expression of adhesion molecules. In the migration assay, human peripheral blood mononuclear cells (PBMC) are placed into the donor compartment of the transwell setup. After 4 h of incubation time, the migrated cells are harvested from the receiver compartment, stained with fluorescent antibodies and counted using flow cytometry. (B) PBMCs were preincubated with S1P for 1.5 h and then transferred into the donor compartment on top of the EC monolayer. S1P inhibits CD3^+^ lymphocyte migration with an EC_50_ of 54.6 ± 49.1 nM, similar to previously published.^22^ (C) In comparison, when EC monolayers were pretreated with pepitem and the PBMC added on top of the peptide-exposed EC, migration of CD3^+^ lymphocytes was inhibited with an EC_50_ of 6.0 ± 6.4 nM. Data are shown as mean ± SD of 3 to 5 independent experiments.

**Table 1:** Overview of the Synthesized Peptide Sequences

Peptide	Sequence^a^	Purity^b^	RT [min]	Calc. [M + H]^+^	Obs. [M + H]^+^	Serum *t*_1/2_ [h]
Pepitem	SVTE **QGAELS** NEER	>95%	18.7	1548.6	1548.6	3.1 ± 0.5
VhTI-pep 1	Ac-EQCKVZCYA **QGAELS** PELLRRCLDNCEK ^c^	>95%	36.3	3248.4	3248.3	>48
VhTI-pep 2	Ac-EQCKVZCYA **QGAEL** -PELLRRCLDNCEK ^c^	>95%	36.4	3161.4	3161.3	>48
VhTI-pep 3	Ac-EQCKVZCY **EQGAEL** -PELLRRCLDNCEK ^c^	>95%	33.4	3219.4	3219.4	>48
VhTI-pep 4	Ac-EQCKVZCY **QGAELS** -PELLRRCLDNCEK ^c^	>95%	25.5	3177.4	3177.4	>48
VhTI-pep 5	Ac-EQCKVZCY **EQGALS** -PELLRRCLDNCEK ^c^	>95%	25.3	3177.4	3177.3	>48
VhTI-pep 6	Ac-EQC **SVT** C- **EQGAEL** -P **SNEER** CLDNCEK ^c^	>95%	26.2	2951.2	2951.0	25.8 ± 9.7
VhTI variant	Ac-EQCKVZCYAQRHSS-PELLRRCLDNCEK ^c^	>95%	29.7	3258.5	3258.5	15.4 ± 12.9

a Amino acids derived from the pepitem/14-3-3ζ sequence are shown in bold letters; norleucine is abbreviated with *Z*; N-termini of the peptides were acetylated (Ac); cysteine connectivity: I–IV, II–III.

b Peak areas were determined by analytical RP-HPLC analysis measuring the absorbance of the compound or impurity peaks at 214 nm (*A*_214_). Half-life (*t*_1/2_) in human serum is shown for VhTI-pep 1–6, as well as the VhTI variant (*n* = 2) and pepitem (*n* = 5) as mean ± SD. RT, retention time in analytical RP-HPLC.

c C-terminal amide.

### 2.2 Sequence Activity Analysis of the Pepitem Molecule and Structure-Based Scaffold Selection

Pepitem has great potency to impede T-cell migration across activated endothelium.^22^ However, as a linear peptide, it is largely unstructured in solution.^27,35^ This limits in silico work on peptide-target interactions as well as rational drug design and development. Moreover, the peptide shows quick metabolic degradation, limiting its in vivo applicability. Hence, the study aimed to establish a rational design approach to capture and stabilize pepitem’s bioactive conformation in a structural scaffold that retains its activity but also transfers enzymatic stability in biological fluids, supporting a broad application scope of these new pepitem-based probes.

To work on this aim, the sequence and structural features of the 14-3-3ζ parent protein of pepitem (PDB: 1QJB) were analyzed to receive information on potential conformations of the peptide fragment (Figure 2A). Interestingly, the pepitem sequence spans residues 28 to 41 of the protein and forms a helix–loop–helix structural motif within the 14-3-3 parent protein. The fragment is surface exposed, but it is not part of the 14-3-3 dimerization interface or of the phosphate-binding pocket. We aimed to identify the bioactive pharmacophore of pepitem with the hypothesis that a minimal active sequence exists within the molecule, in accordance with the reported activity for the full-length 14-3-3ζ protein isoform.^27^ The sequence of pepitem is unique for the 14-3-3ζ isoform among the seven human 14-3-3 proteins (Figure 2B). The N-terminal segment showed moderate conservation of residues between the isoforms (e.g., 28–31), the central amino acids (e.g., 29–34) revealed considerable variability, and the C-terminal segment had several highly conserved residues, i.e., Leu^36^ and Glu^39^ to Arg^41^. To gain more insights into sequence conservation of the peptide, we investigated the 14-3-3ζ proteins among species of the phylum chordata. A blast analysis identified 290 sequences from as many different species (Table S2). Interestingly, the pepitem sequence has highly conserved residues within the chordata species, especially residues Val^29^ to Glu^31^, Leu^36^, and Glu^39^ to Arg^41^ (Figure 2C). In addition, further grouping of the obtained sequences into mammals, birds, reptiles, amphibians, and fish revealed that the N- and C-terminal segments are highly conserved within all species, and very little sequence variation, if at all, was detected in mammals, birds, reptiles, and amphibians. However, the loop sequence in fish possesses a highly variable amino acid configuration, especially the motif from positions 5 to 9 (Figure S4). Considering the conformation of the pepitem sequence in the 14-3-3ζ protein in combination with sequence diversity over evolution, it was rational to assume that the alpha helical segments shape the high integrity of the helix–loop–helix peptide, whereas the surface-exposed residues of the loop region in the 14-3-3ζ protein preserve a bioactive epitope. To test this hypothesis, we performed a fragment-based approach to test truncated variants of pepitem for bioactivity. Pepitem fragments, resembling the helix motifs (truncated pep-1, truncated pep-3) as well as the loop region (truncated pep-2) (Table S3, Figure S5A–C) were analyzed in a concentration response experiment between 0.1 and 10 μM (Figure S5D). Truncated pep-1 and truncated pep-3 showed no reduction of cell migration, with the exception of truncated pep-3 at the highest concentration, with a trend for reduction of migration by 20.1 ± 14.7%. In comparison, the truncated pep-2 decreased CD3^+^ cell migration, for example, with a maximum of 32.1 ± 18.9%, which is similar to pepitem (30.3 ± 8.0%) at the same concentration (Figure S5D). The data further supported the finding that one possible bioactive conformation is conserved within the loop segment of pepitem. Since the conformation of the pepitem sequence in the 14-3-3ζ parent protein (X-ray crystal structure) may not resemble the conformation of pepitem in the target-bound state, but a similar or another bioactive conformation, we further aimed for a peptide design allowing chemical and conformational flexibility for the loop segment, which we considered a key to success for the molecular grafting work.

**Figure 2:**
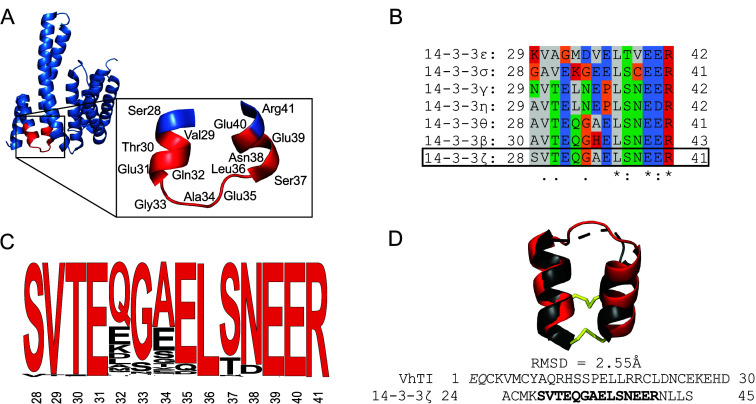
**Molecular analysis of the pepitem structure and selection of VhTI as a structurally similar scaffold peptide**. (A) The structure of the 14-3-3ζ monomer (derived from PDB: 1QJB) is shown, with a zoomed-in view on the region corresponding to amino acids 28–41 (highlighted in red), corresponding to the sequence of pepitem (14-mer). The pepitem sequence builds a helix–loop–helix motif in the protein; the amino acids are labeled in the zoom-in. (B) The sequence alignment shows the seven human 14-3-3 protein isoforms of the region corresponding to the pepitem sequence in the zeta isoform, where the unique amino acid sequence for pepitem is found. The positions of the first and last amino acids of the sequence in the parent protein are labeled. Positively charged amino acids are highlighted in red, negatively charged residues are shown in blue, polar uncharged amino acids in green, amino acids with hydrophobic side chains in gray, and glycine, cysteine, and proline in orange. The human 14-3-3 protein isoforms show several conserved residues in the N-terminal region. (C) A sequence similarity analysis was conducted for 290 chordata species (based on the 14-3-3ζ protein isoform), and a sequence logo was prepared showing the corresponding pepitem region, with the pepitem sequence depicted in red. The amino acid residues are labeled based on their position in the human protein. (D) Alignment of the VhTI peptide with the 14-3-3ζ protein fragment. The published structure of the VhTI peptide (gray, PDB: 2CMY) was aligned to the extended 14-3-3ζ protein fragment (residues 24–45) comprising the pepitem epitope (red, PDB: 1QJB). The helical peptide framework of the two peptides was aligned using the cealign command in PyMOL resulting in a calculated RMSD of 2.55 Å. The loop sequence proved to be more flexible in our alignment analysis than the helical segments. The sequences used for the alignment are shown below. The numbers before and after the sequence state the position of the first and last amino acids in the full-length sequence of the VhTI peptide and the 14-3-3ζ protein monomer. Some amino acids of the VhTI peptide were not resolved in the PDB structure and are marked in gray in the sequence below. The disulfide bonds of the VhTI peptide are shown in yellow.

It appeared rational to maintain these secondary structures on the peptide level to promote activity. Therefore, we aimed to find a suitable natural peptide scaffold for embedding the pharmacophore sequence in a bioactive conformation, applying a structure-inspired molecular grafting approach. To identify and select structurally analogous molecules, a structure-based database search was performed using the PDB database. For this, we derived the structure of the pepitem sequence within the 14-3-3ζ parent protein (PDB: 1QJB). The model was utilized as the query to conduct a structure-similarity search in the PDB database using the advanced search option. This search algorithm compares electron density volumes derived from the protein structures to find similarities in biomolecules.^36^ The initial search yielded 2,062 structures and was filtered to retain peptides and small proteins, reducing the data set to 286 entries. Further refinement by manual curation to select cysteine-rich peptides containing at least two cysteine residues and sequences ranging from 10 to 50 amino acids in length. Redundant and duplicate structures were excluded, resulting in 110 unique structures, which were classified into 11 groups for further analysis: α-hairpinins, Bowman-Birk inhibitor peptides, β-hairpinins, conotoxins, other cyclic peptides with a single disulfide bond, other cyclic peptides with two disulfide bonds, endothelin-derived peptide, enterotoxin-derived peptides, knottins, scorpion toxins and an additional category named “others” combining those that did not fit into the predefined groups. The list of all identified peptides in the search is provided in Table S4 and the pie statistic are shown in Figure S6A. Based on the visual inspection of the peptide structures across the different groups, most candidates had a moderate global structural similarity to the helix–loop–helix conformation of pepitem. They were identified as hits based on similarity in electron density volumes by the search algorithm,^36^ but did not resemble the shape of the helix–loop–helix motif. Similarly, prototypic natural scaffold peptides (kalata B1, MCoTI-II, and theta-defensins), which were not identified in the initial PDB search, do not exhibit the desired conformation. For visualization, Figure S6B shows representative structures for the groups with an alignment to pepitem, highlighting only limited conformational similarity. However, our PDB search identified two α-hairpinin peptides that, like the pepitem structure, adopt a helix-turn-helix conformation (Figure S6C, Figure 2D). This prompted a focused evaluation for structural similarity of pepitem with other members of the α-hairpinin peptide family. For this, we evaluated five representatives of the α-hairpinin peptide family with PDB structures deposited in the public domain. First, we determined the overall structural alignment of the candidate molecules to the 14-3-3ζ fragment containing the pepitem sequence. Indeed, the alignment indicated conformational fit of several of the analyzed peptides to pepitem (Table S5, Figure S6C). As our aim was to design peptides that still had flexibility in the loop sequence to allow for the mapping of activity of different loop conformations, we refined the alignment by constraining the alignment to the helical framework only, giving space for flexibility in the loop region. We used the two alignment algorithms, “super” and “cealign,” in PyMOL to identify the most suitable scaffold (Table S6). The virus-derived BRSV-peptide was not further considered because of its reported immunodominance and strong antigenic properties. Overall, the *Veronica hederifolia* trypsin inhibitor (native VhTI, P85981 TI_VERHE, PDB: 2CMY) emerged as the best match, displaying a confident conformational alignment with an RMSD of 2.55 Å (Figure 2D, Table S5) when the helical framework was aligned and appeared as the most suitable scaffold. Native VhTI adopts a helix–loop–helix motif with a two-disulfide framework connecting the helical segments. The disulfides serve as natural restrictors for flexibility, comparable to helix staples, leading to high stability of the entire molecule.^37,38^ Consequently, VhTI was selected to proceed with structure-inspired molecular grafting as it obtained a superior conformational fit for the bioactive peptide.

### 2.3 Rational Design of Modulators for Lymphocyte Transendothelial Migration

Prediction models of chimeric probes using AF2 were prepared^33,34^ by combining the VhTI scaffold derived from structure-inspired selection and the identified bioactive amino acid motif. The concept of the approach is depicted in Figure 3A. For the scaffolding peptide, we used a previously published variant, spanning residues 5–31 of the native VhTI peptide.^37^ The loop segment of pepitem (EQGAELS^4–10^) was embedded in the loop region of the VhTI scaffold with different configurations, replacing residues 9–14 in VhTI. In addition, three peptides containing the full-length pepitem sequence were designed, where the peptide’s helical motifs were incorporated in the helical region of VhTI. The primary sequence information for the designs is provided in Tables 1 and S6. All models, except VhTI-pep 9, indicated that the peptides adopt the helix–loop–helix motif, supporting the VhTI scaffold-based grafting approach (Figure S7). To further analyze the predicted structures, model-to-structure alignment experiments were performed. First, the alignment of the modeled VhTI variant with the published structure for the VhTI peptide (PDB: 2PLX) resulted in a good fit with a calculated RMSD of 0.51 Å. Next, the modeled structures of the probes VhTI-pep 1–9 were aligned to the published VhTI structure. VhTI-pep 1–7 showed a good fit with RMSD < 1.5 Å, and VhTI-pep 8 with RMSD of 1.8 Å (Table S7). Overall, the analysis indicated that the helical elements aligned similarly in all the peptides, providing a robust framework to home the pharmacophore of pepitem. A detailed molecular inspection verified that slightly varying positions and length of the epitope facilitated flexible positioning of side chain functionalities, allowing mapping of chemical space in the various modeled peptides (Figure 3B). Based on the calculated RMSD values and the detailed inspections, six out of the nine modeled peptides (VhTI-pep 1–6), as well as the VhTI scaffold molecule, were selected for solid-phase peptide synthesis (SPPS). Norleucine, a bioisostere to methionine, was introduced to eliminate the oxidation-prone methionine in position 6 of VhTI (VhTI-pep 1–5, VhTI variant). The VhTI-peps were N-terminally acetylated and C-terminally amidated to provide protection against exoproteases. The correct disulfide connectivity was ensured through a directed folding strategy. The synthesized and folded peptides were purified, and mass spectrometric analysis confirmed the correct masses (Figure S2B–G).

**Figure 3:**
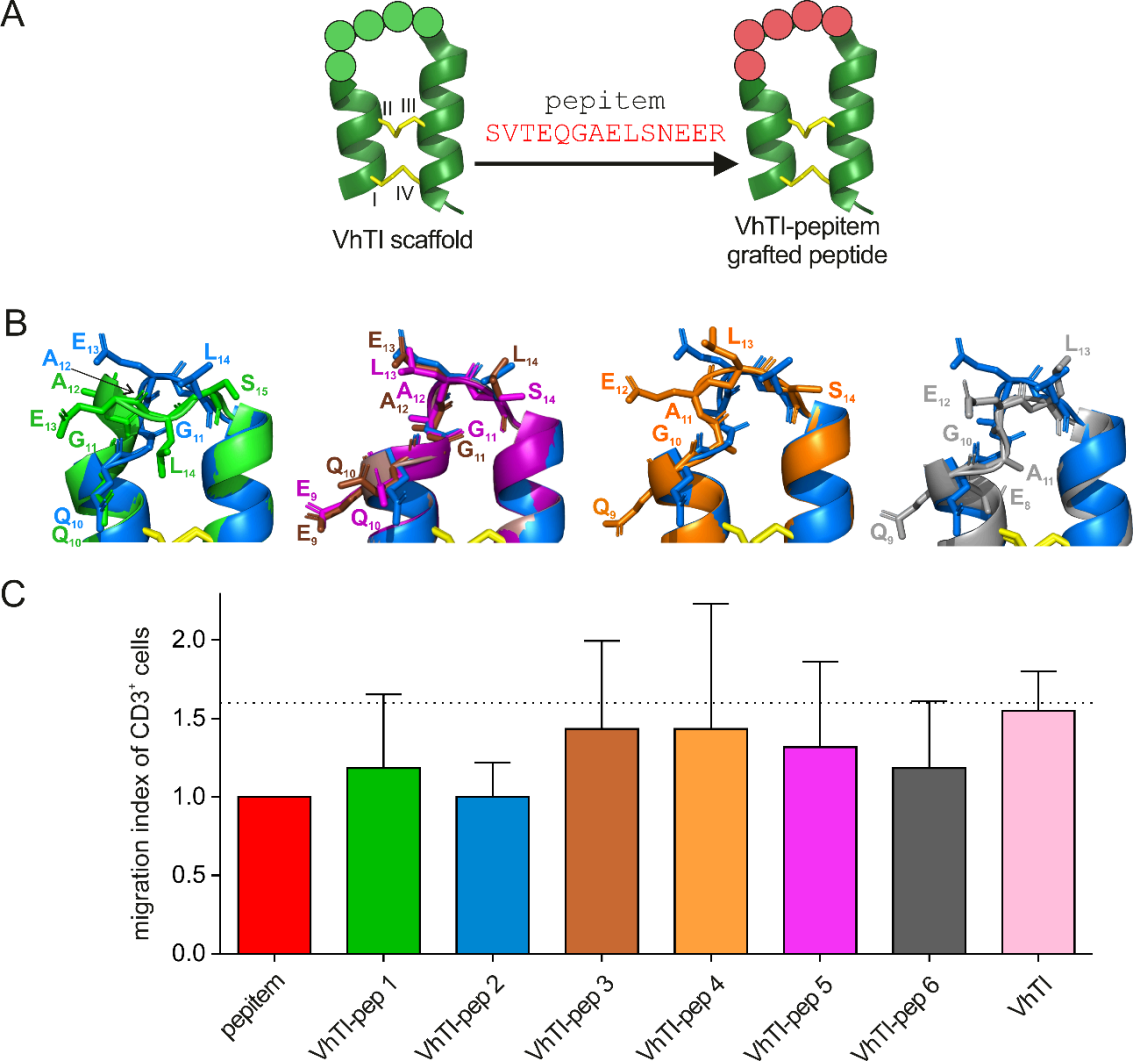
**Design and bioactivity of grafted VhTI-pepitem probes**. (A) Illustration of the principle of peptide molecular grafting. A variant of the VhTI peptide is applied as the scaffold molecule. A certain sequence derived from the bioactive pepitem is incorporated into the loop region of VhTI. The scaffold peptide (PDB: 2PLX^37^) is shown in green with the disulfide bonds in yellow; the cysteine residues are labeled as I to IV (cysteine connectivity is I–IV, II–III) and an example of a grafted sequence of pepitem is highlighted in red. (B) The structure of the peptides was predicted using AF2 together with a workflow for modeling of cyclic peptides^33,34^ (Figure S7). The VhTI scaffold adopts a very similar conformation but allows for the display of different loop motifs based on the amino acid configuration. The view shows an overlay of the different models in the grafted loop region to illustrate the side chain orientation and positions for the compared models for VhTI-pep 1 (green), VhTI-pep 2 (blue), VhTI-pep 3 (brown), VhTI-pep 4 (orange), VhTI-pep 5 (magenta) and VhTI-pep 6 (gray). The grafted amino acids are labeled with one-letter codes, and their position in the corresponding peptide is indicated. (C) The effect on the migration of CD3^+^ cells was assessed for the peptides. A migration index relative to the migrated cells in the pepitem-treated control group was calculated (pepitem-treated samples were set as 1.0 in order to compare the effects of pepitem with the designed peptides and the empty VhTI scaffold). The dotted line represents the fraction of migrated cells in the untreated control. Data are shown as mean ± SD for 4–6 independent experiments.

To validate the design approach, human serum stability assays as well as bioactivity assessments were conducted. In comparison to the reference compound pepitem with a serum half-life of *t*_1/2_ = 3.1 ± 0.5 h, all probes were highly stable with *t*_1/2_ > 48 h for VhTI-pep 1–5, *t*_1/2_ = 28.7 ± 9.7 h for VhTI-pep 6 and *t*_1/2_ = 15.4 ± 12.9 h for VhTI (Table 1, Figure S8A). To evaluate the bioactivity of the grafted peptides, the inhibition of T-lymphocyte migration was studied in a transwell migration assay for the peptides in two concentrations. Compared to pepitem, VhTI-pep 1 and VhTI-pep 3–6 had reduced bioactivity, as indicated by an increase in the migration index relative to pepitem (Figure 3C). At 0.1 μM, VhTI-pep 3–5 displayed only weak effects on CD3^+^ lymphocyte migration. VhTI-pep 1 and VhTI-pep 6 reduced CD3^+^ cell migration by 25.6 ± 6.4% and 27.0 ± 9.7%, respectively (Table 1). The VhTI scaffold itself had no effects, equal to untreated control conditions. VhTI-pep 2 outperformed all other analogs with similar activity (28.7 ± 6.4%) compared to pepitem (26.7 ± 5.3%; Figure 3C; Table 1). In addition, effects on migration of CD8^+^ and CD4^+^ lymphocytes as well as memory phenotype cells (CD4^+^/CD8^+^ and CD45RO^+^ cells) were observed, with a similar trend as for CD3^+^ cells (Table 1). VhTI-pep 2 inhibited CD4^+^ cell migration at a concentration of 0.1 μM by 34.3 ± 8.0%, whereas VhTI-pep 1 only inhibited this subset by 23.5 ± 9.7%. Even though both VhTI-pep 1 and 2 displayed good activity, VhTI-pep 2 was the superior candidate, taking into account the consistent effects toward all tested T-lymphocyte populations. All probes had no recorded cell cytotoxicity on the EC up to 1 μM (Figure S8). In summary, the serum stability of all tested probes was increased in comparison to pepitem. VhTI-pep 2 exhibited the best activities in the migration assay (Table 2, Figure 3C) and was selected for further structural and pharmacological validation studies.

**Table 2:** Bioactivity Characterization of Pepitem, Grafted VhTI-pep 1-6 and VhTI Variant^a^

% Inhibition of cell migration compared to control						
Peptide	Conc. [μM]	CD3^+^	CD4^+^	CD8^+^	Memory CD4^+^	Memory CD8^+^
Pepitem	1	38.7 ± 7.4	33.4 ± 7.7	34.3 ± 8.2	22.2 ± 7.0	27.7 ± 8.1
	0.1	26.7 ± 5.3	26.2 ± 7.2	28.1 ± 4.8	32.1 ± 10.7	17.8 ± 8.5
VhTI-pep 1	1	33.4 ± 8.5	30.3 ± 14.7	38.4 ± 2.4	39.5 ± 11.6	29.6 ± 6.7
	0.1	25.6 ± 6.4	23.5 ± 9.7	34.6 ± 8.0	15.1 ± 14.2	21.2 ± 3.1
VhTI-pep 2	1	37.2 ± 6.8	33.2 ± 6.1	41.7 ± 8.5	31.5 ± 10.4	21.9 ± 8.1
	0.1	28.7 ± 6.4	34.3 ± 8.0	37.0 ± 6.4	34.4 ± 16.7	23.0 ± 6.9
VhTI-pep 3	1	4.6 ± 10.6	10.8 ± 10.1	14.0 ± 13.8	11.7 ± 10.6	3.0 ± 11.6
	0.1	1.5 ± 7.6	–8.9 ± 18.1	10.4 ± 11.2	–8.7 ± 3.4	3.9 ± 9.7
VhTI-pep 4	1	24.4 ± 8.0	24.5 ± 5.9	26.0 ± 10.7	27.2 ± 7.8	19.7 ± 7.9
	0.1	16.7 ± 11.0	16.5 ± 7.3	19.5 ± 20.2	12.9 ± 11.0	27.9 ± 3.8
VhTI-pep 5	1	28.4 ± 7.4	33.5 ± 5.7	24.8 ± 12.8	28.6 ± 8.8	14.0 ± 13.4
	0.1	18.0 ± 6.2	16.2 ± 7.7	20.8 ± 6.8	11.5 ± 13.6	4.7 ± 10.1
VhTI-pep 6	1	14.9 ± 11.6	14.6 ± 8.7	1.6 ± 7.8	14.7 ± 7.6	0.6 ± 6.1
	0.1	27.0 ± 9.7	19.5 ± 9.9	28.0 ± 8.2	24.6 ± 11.6	23.2 ± 4.3
VhTI variant	1	–5.2 ± 10.7	–6.7 ± 7.0	–2.8 ± 15.8	–11.6 ± 13.2	–11.6 ± 7.2
	0.1	–3.9 ± 7.0	–7.8 ± 9.4	1.6 ± 8.5	–3.5 ± 2.4	4.7 ± 7.4

a Inhibition is expressed as % inhibition normalized to the untreated control (= maximum migration observed under test conditions). Data are shown as mean ± SEM of 4-6 experiments.

### 2.4 In-Solution Structure of VhTI-pep 2

To gather more information on the structural conformation of bioactive VhTI-pep 2, it was subjected to structural analysis by in-solution NMR spectroscopy (Figure 4A). ^**1**^H-^**1**^H homonuclear and ^**1**^H-^**13**^C/^**15**^N heteronuclear 2D experiments were recorded at natural abundance, and the data were of exceptional quality with sharp lines and excellent signal dispersion, consistent with a highly ordered structure. Complete resonance assignments were achieved for all backbone and side chain resonances, and no additional resonances suggesting minor conformations were observed (Figure S9A–D). The Pro residue was in a trans conformation based on both NOE patterns and ^**13**^C chemical shifts. The NMR data were used to derive structural restraints, including interproton distances, backbone and side chain dihedral angles, and hydrogen bonds. These allowed the calculation of the solution structure of VhTI-pep 2 using simulated annealing approaches and energy minimization in explicit water.^40^ A structural ensemble of the 20 best conformations was produced (Figure S9E), and structural statistics were generated (Table S8). The backbone and side chain RMSDs were 0.31 Å and 0.99 Å, respectively, resulting from an unusually large number of restraints for such a small peptide, supporting a structure of excellent quality. The structure was, as predicted, a helix–loop–helix fold stabilized by the disulfide bonds and by a large number of backbone hydrogen bonds and side chain interactions. The latter included hydrophobic packing, *e.g.,* involving Tyr^8^, Leu^18^_,_ and Leu^22^, as well as ionic interactions between the negatively charged Glu^16^, Asp^23^_,_ and Glu^26^, which were perfectly interspersed by the positively charged Arg^19^, Arg^20^_,_ and Lys^27^ on the C-terminal helical surface (see Table 1 for primary sequence information). Interestingly, the grafted sequence was partially located in the loop segment; in detail, Gln^10^ was attributed to the helical structure, while GAEL^11–14^ contributed to the loop segment. Measurements suggested that the QGA^10–12^ sequence contained the most dynamic residues in the peptide (Figure S9F). Therefore, these residues might adopt flexible conformations upon binding to target proteins. Importantly, residues Gln^10^, Glu^13^ and Leu^15^ had side chain orientations toward the exterior space, which would allow for interactions with possible target proteins. An alignment of the AF2 predicted and obtained NMR structure of VhTI-pep 2 resulted in a RMSD of 1.07 Å. It was noted that the side chain orientation of the loop (QGAEL^10–14^) was correctly predicted and overlaid almost perfectly in both models. This excellent fit between the modeled and the experimentally obtained structure further supported the approach to select natural peptide scaffolds by conformational fit for peptide pharmacophore embedding (Figure 4B).

**Figure 4:**
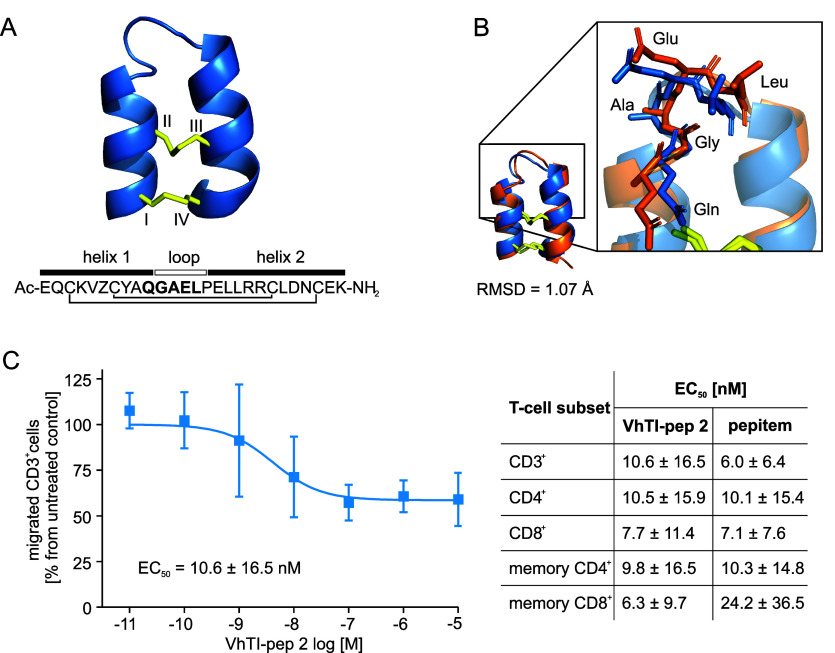
**Characterization of VhTI-pep 2**. (A) Cartoon representation of the VhTI-pep 2 in solution structure determined by NMR confirms the helix–loop–helix conformation of the designed peptide. The cysteine residues are labeled in the structure (I–IV). The peptide sequence is shown below and the cysteine connectivity is indicated with black lines. The helix segments (black box) and the loop (empty box) are indicated above the sequence. (B) An overlay of the experimentally determined structure (blue) with the modeled structure using AF2 (orange) resulted in a calculated RMSD of 1.07 Å. The zoom-in shows a strong alignment of the grafted sequences between the structures. The amino acid side chains are shown in stick representation and are labeled with the three-letter code. (C) A concentration–response experiment was conducted for VhTI-pep 2. The peptide inhibited CD3^+^ lymphocyte migration with an EC_50_ 10.6 ± 16.5 nM. The inserted table shows the measured EC_50_ values for inhibition of migration of other T-cell subsets, including CD4^+^ cells EC_50_ = 10.5 ± 15.9 nM, CD8^+^ cells EC_50_ = 7.7 ± 11.4 nM, memory CD4^+^ cells EC_50_ = 9.8 ± 16.5 nM and memory CD8^+^ cells EC_50_ = 6.3 ± 9.7 nM (Figure S10) for VhTI-pep 2 as well as, in comparison, the obtained EC_50_ values for pepitem for these subsets (graphs shown in Figures 1C and S3). Data are shown as mean ± SD for 4 independent measurements.

### 2.5 VhTI-pep 2 Peptide Is a Potent Modulator of Lymphocyte Migration from Healthy Donors and Treatment-Naïve Patients Diagnosed with Multiple Sclerosis

Since only two peptide concentrations (0.1 and 1 μM) were tested during the first bioactivity screen, we pursued a full concentration–response of the VhTI-pep 2 lead peptide. VhTI-pep 2 inhibited CD3^+^ cell migration with an EC_50_ of 10.6 ± 16.5 nM and CD4^+^ or CD8^+^ lymphocytes with an EC_50_ of 10.5 ± 15.9 nM and 7.7 ± 11.4 nM, respectively. The migration of memory T-cell phenotype cells was inhibited in the low nanomolar range as well (Figures 4C and S10). This activity was similar to the EC_50_ obtained for linear pepitem for all evaluated T-cell populations; for example, pepitem inhibited CD3^+^ cell migration with an EC_50_ of 6.0 ± 6.4 nM (Figures 1C and S3). In addition, the maximal effect with which VhTI-pep 2 inhibited cell migration in comparison to pepitem was 39.3 ± 8.7% (pepitem 31.0 ± 18.6%) for CD3^+^-, 39.9 ± 12.5% (32.7 ± 14.0%) for CD4^+^- and 40.0 ± 2.8% (30.6 ± 18.4%) for CD8^+^-lymphocytes (Figures 1C, 4C, S3, and S10). These data highlight the similar efficacy of both peptides in inhibiting the migration of the different T-cell populations. Taken together, the activity of VhTI-pep 2 highlights successful grafting of the pharmacophore of pepitem.

So far, all migration assays have been conducted using PBMCs isolated from healthy donors. Consequently, we sought to evaluate the novel peptide within a more clinically relevant context. Samples from patients recently diagnosed with MS, who had not undergone any prior immunosuppressive therapy, were utilized for this purpose. These samples might present activated states of the different immune cell populations, such as an increase in antigen-reactive T-cells^41^ or a change in total cell count (e.g., an increase in Th1 or Th17 lymphocytes^42^) compared to healthy subjects. Therefore, utilizing the in vitro assay, we again observed the activity of the peptides on different disease-relevant T-cell subsets, including memory T-cells and the known disease drivers Th1 and Th17 (Figure 5). Migration was significantly reduced by VhTI-pep 2 for CD3^+^ (*p* = 0.0008) and CD4^+^ (*p* = 0.0005) cell populations. For the CD8^+^ cell population, a positive trend was visible, although the limited number of cells in the patient samples did not allow us to test for significance. Interestingly, only VhTI-pep 2 and not pepitem significantly inhibited the migration of Th1 lymphocytes by 34.3 ± 11.4% (*p* = 0.048). Th17 lymphocytes did not achieve high total counts in these patient samples, restricting reliable experiments in this study.

**Figure 5:**
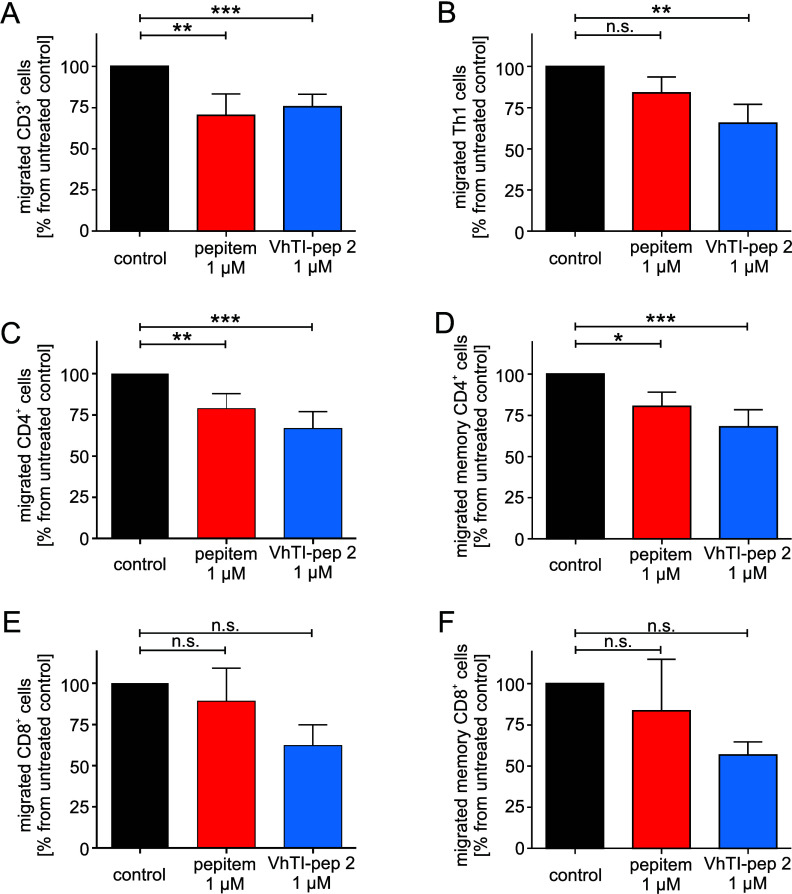
**Activity of the novel VhTI-pep 2 and pepitem on patient lymphocytes**. PPBMCs were isolated from the blood of MS patients with a very recent disease flare receiving no immunosuppressive or immunomodulatory treatment in the past. The effect of the linear pepitem and VhTI-pep 2 was tested for the T-cell populations (A) CD3^+^, (B) Th1, (C) CD4^+^, (D) memory CD4^+^, (E) CD8^+^, and (F) memory CD8^+^ cells. Data are shown as mean ± SD for 4 independent measurements. Statistical analysis was performed on the level of total counted cells using repeated measures one-way ANOVA with Dunnett’s post hoc test. Statistical significance is indicated as n.s. (not significant), * *p* < 0.05; ** *p* < 0.01; *** *p* < 0.001.

In summary, the designed novel VhTI-pep 2 peptide proved to have similar bioactivity in comparison to linear pepitem in the in vitro migration assays. In particular, its activity on patient-derived Th1 and memory T-cell migration highlighted the effect of the peptide in a disease setting. Hence, VhTI-pep 2 can be considered a promising therapeutic lead for efficacy studies in models where T-lymphocytes are crucial disease drivers.

### 2.6 Prediction of VhTI-pep 2 - Target Protein Interaction

VhTI-pep 2 is a potent inhibitor of lymphocyte migration and a lead for further development. Importantly, the in-solution structure of the peptide offers new insights into the bioactive conformation of the pepitem epitope and a model for the prediction of molecular interactions with target proteins. While pepitem interacts with CDH15 to modulate immune cell migration,^22^ the neural cell adhesion molecule (Ncam-1, CD56) has been proposed as a target protein for pepitem in regulating bone formation, and in silico modeled molecular interactions between pepitem and CD56 were modeled previously.^35^ To investigate any potential binding or interaction sites between VhTI-pep 2 and Ncam-1 and CDH15, we generated 3D models of the corresponding complexes using AF3.^43^ To visualize the 3D structures, we used visual molecular dynamics, coloring the models according to the plDDT scores to highlight regions of structural confidence. The Ncam-1 model had good overall pIDDT scores of >80. We compared the model with a reported X-ray structure (PDB: 3MTR) and found that the AF3 model aligned very well with the experimentally determined structure. Ncam-1 is structurally composed of five Ig-like (denotade asIg-1 to Ig-5) and two fibronectin-III-like (FN1 and FN2) domains. Modeling the interactions between pepitem and Ncam-1 (Figure S11), we could see binding of pepitem to the FN2 domain as proposed previously.^35^ Modeling the interaction between VhTI-pep 2 and Ncam-1 suggested interactions with the FN1, Ig4 as well as Ig5 domains, with the best-scoring model for the interaction with the Ig5 domain of CD56 (Figure 6A).^44^ To refine this interaction, we performed a follow-up experiment, focusing specifically on the FN1, Ig4, and Ig5 domain fragments to improve model confidence through increased sampling. In this focused analysis, the best-scoring model confirmed interaction with the Ig5 domain of VhTI-pep 2 with higher resolution and confidence and a local interaction score (LIS) of 0.47 (Figure 6B). The LIS value can range from 0 to 1, where small interaction binding interfaces or flexible binding sites typically show moderate LIS values (0.4–0.8), balancing stability and flexibility of the involved domains. The calculated LIS score improved for the targeted prediction using the isolated domains, indicating that the domain flexibility in the full-length proteins limited global accuracy. In general, the experimentally obtained LIS values were in an acceptable range, similar to what was reported for training sets in the original work.^44^ We saw that the scaffold-embedded pepitem pharmacophore is oriented toward the protein side, forming several polar and hydrophobic interactions with the protein (Figure 6C–E), modeled with the protein–ligand interaction profiler (PLIP) online tool.^45^ The PLIP algorithm detects noncovalent interactions between input proteins using interaction characteristics and geometric rules for interactions known from literature. The interactions of VhTI-pep 2 come from the QGAEL^10–14^ sequence in the loop region, in particular E^13^, as well as from amino acids from the scaffolding peptide, namely Glu^1^, Val^5^, Tyr^8^, Leu^18^_,_ and Leu^22^ (Figure 6D,E, and Table S9). Interestingly, the VhTI-pep 2 binding site partially overlapped with an interface for Ig5 and FN1, important for the homodimeric interactions. There are a few peptides reported to target Ncam-1 Ig-like domains, mostly domains I to III, but subdomain Ig5 is not among them.^46,47^ In the case of CDH15, the best-scoring models of the interaction of linear pepitem (Figure S11B) and full-length VhTI-pep 2 (Figure S12A) both resulted in ambiguous predictions, with a slight preference for binding to the N-terminal domain, in particular the extracellular 1 domain of the protein. This preference was further examined by modeling of the isolated CDH15 fragment, providing an LIS score of 0.51 (Figure S12B). The fragmented pairing indicated interaction signals within the N-terminal region. These models provide a basis for identifying potential interaction interfaces between VhTI-pep 2 and both Ncam-1 and CDH15. Table S10 provides an overview of the AF3-predicted protein and peptide residues involved in the peptide–protein interaction in the different models.

**Figure 6:**
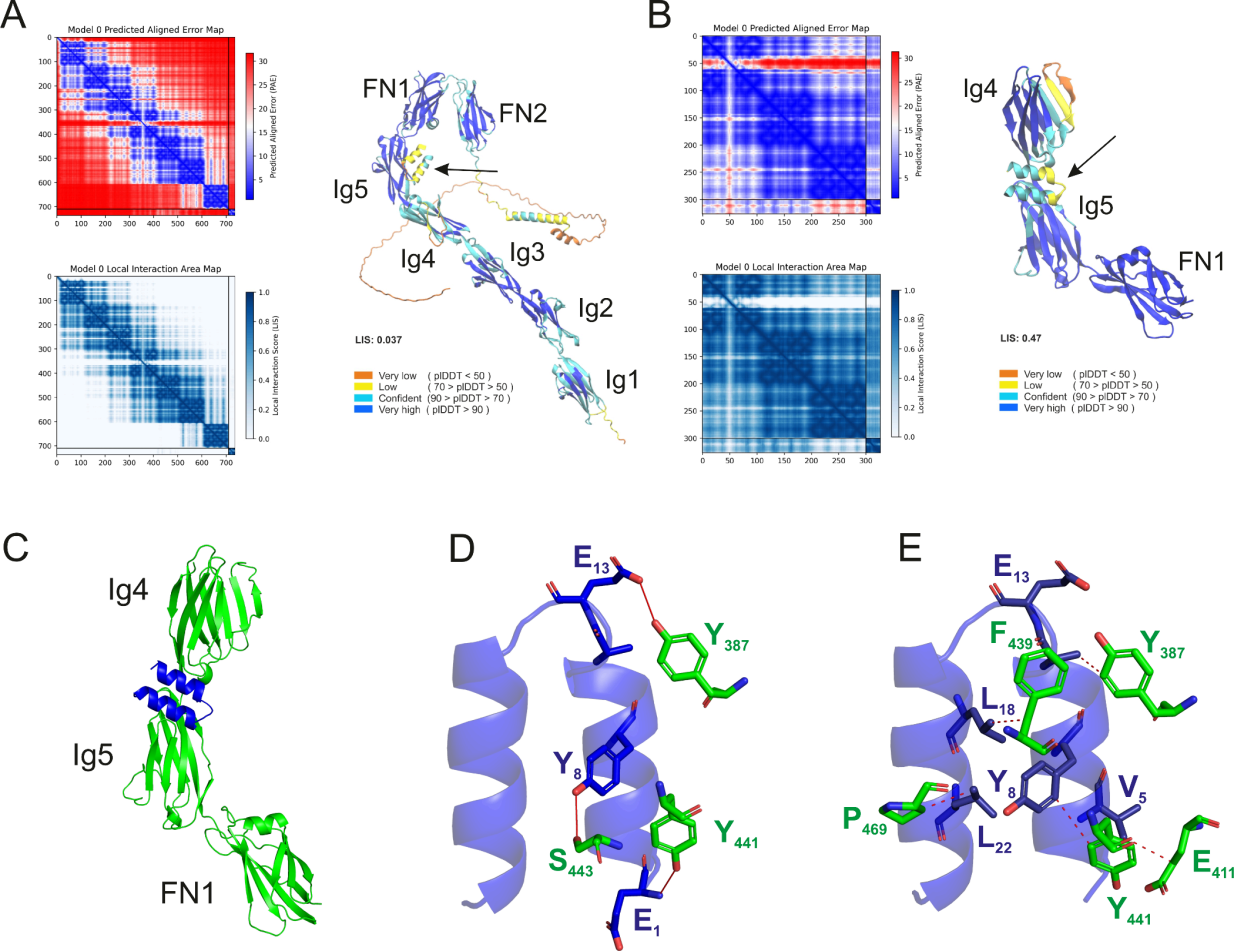
**Peptide–protein interaction models. Interactions between VhTI-pep 2 and Ncam-1 were modeled using AF3**. (A) The interaction was modeled using the full-length CD56 protein and (B) a protein fragment. Ncam-1 is built by five Ig-like domains labeled as Ig1–5 and two fibronectin-III-like domains (FN-1 to 2). For all models, the Predicted Aligned Error (PAE) matrix for the X-Y complex is shown, which highlights regions of higher structural uncertainty. The local interaction area visualization shows regions of high Local Interaction Score (LIS) within the complex. The 3D structure of the X-Y complex is color-coded by plDDT to indicate local structural confidence. The arrows indicate the position of the peptide in the model. (C) Zoom in on the interaction site of VhTI-pep 2 (blue) and Ncam-1 (green). Using the PLIP online tool^45^ we predicted the interactions of VhTI-pep 2 with the protein. (D) Three hydrogen bonds were predicted between the peptide and Ncam-1. The hydrogen bonds are presented as red lines between the respective atoms. (E) A total of six hydrophobic interactions between the VhTI-pep 2 and the protein were predicted. The hydrophobic interactions are shown as red dashed lines connecting the respective atoms of the amino acid residues involved. Details on the predicted interactions can be found in Table S9. VhTI-pep 2 is shown in blue in the cartoon representation, with amino acids contributing to interactions additionally highlighted in the stick representation. For the Ncam-1 protein, only amino acid residues involved in interactions are shown in green. All amino acids involved in the respective interactions are labeled in one-letter code with their respective position in the peptide or Ncam-1 protein (the full-length protein was used as the reference for numbering the protein residues).

In summary, we introduced a new workflow for a structure-based grafting strategy, delivered proof-of-concept data with VhTI-pep 2, and showcased peptide–protein interaction predictions with the stabilized probe.

## 3 Discussion and Conclusions

Therapeutic peptides populate an important space in the pharmaceutical market, with over 90 peptide therapeutics that have been approved to date.^48,49^ With the rapid success of GLP-1 receptor agonists, peptides are emerging as the next generation of blockbuster drugs and are on their way to strip off PD-1 antagonists as best-selling drugs worldwide by 2025.^48,50^ Here, we introduce a novel approach for engineering stabilized peptides through structural scaffold matching. A proof-of-concept was demonstrated using pepitem, an endogenous modulator of immune cell migration. The pharmacophore of pepitem was predicted by sequence-activity and structural homology analysis. Guided by structural mining of the protein database and matching of the endogenous pepitem conformation derived from the parent 14-3-3ζ protein, the bioactive sequence was embedded into the conformationally compatible VhTI peptide, creating stabilized peptide analogs VhTI-pep 1–6. The culminating lead VhTI-pep 2 exhibited comparable activity to pepitem in T-lymphocyte transendothelial migration assays. We obtained a highly stabilized and high-resolution NMR structure of VhTI-pep 2 revealing the expected loop-helix-loop motif and enabling predictions of its interactions with its endogenous protein targets, CDH15 and Ncam-1. This approach not only provided new molecular insights into the SAR of pepitem but also validated a rational structure-guided scaffold matching approach to design stabilized peptides with predictable activity.

Despite some exquisite advantages of peptide therapeutics, such as high potency and exquisite target selectivity, they often have limited metabolic stability in biological fluids and are rapidly cleared through glomerular filtration and excretion. PEGylation or lipidation strategies were introduced overcoming the renal clearance limitation of peptides. Different approaches have been pursued for protecting peptides from proteolysis,^48,50^ one of them being molecular grafting.^51^ The approach has been used to design peptides for a wide variety of indications, including cancer,^52^ obesity^51^ or pain^31^ and in some instances resulted in gut activity^53^ or oral availability.^54^ With some exceptional examples of very long sequences,^55^ short epitopes of less than 10 residues are typically used for molecular grafting.^28^ Recently, a so-called ‘plug-and-play’ approach introduced a methodology avoiding tedious folding of cyclotide probes by chemical ligation of even larger sequences on the prefolded prototypic kalata B1.^55^ Similarly, the implementation of stabilizing β-turn motifs in cyclic cystine-rich peptides improved the scaffold’s tendency to fold into its native-like conformation.^56^ Despite numerous advancements directed to secure success, molecular grafting remains an experimental ligand-based approach marked by a high failure rate.^28^ Aiming to obtain peptides in the active conformation to improve successful peptide design, the use of anchor residues to lock the grafted peptide into its active conformation has been emphasized.^57^ Similarly, tools, such as LoopGrafter^58^ or advanced protocols^59^ have been developed to guide successful grafting approaches. Still, the process of scaffold selection has been largely underexplored in the past, with insufficient emphasis on rational approaches to identify and use conformationally similar scaffold peptides. This study proposes to integrate rational scaffold selection together with predictive modeling prior to peptide synthesis. Stabilizing the bioactive conformation of the target sequences utilizing peptide scaffolds with conformational fit to the epitope enhances the likelihood of maintaining activity of such chimeric molecules. While protein or peptide similarity analysis often takes advantage of the vast information available for the primary sequence level, identifying appropriate molecules for embedding bioactive epitopes requires prioritizing 3D structural compatibility. Sequence-based searches often fail to capture structural congruence; therefore, tools that assess 3D similarities are required. Recent computational advancements have accelerated such structure-based similarity searches. Foldseek^60^ and Dali (distance matrix alignment)^61^ enable rapid structure-based library searches. Foldseek, for example, translates the structural information into a sequence-like format, allowing alignment-based comparisons of structures.^60^ We put Foldseek as well as Dali to the test to identify suitable scaffold proteins for pepitem. However, neither of the tools could successfully identify stabilized peptide structures, which could serve as scaffolds for grafting studies (data not shown). These algorithms were mainly developed for the search and comparison of large globular protein structures. For instance, we found Dali requires query structures longer than 30 residues,^61^ making these tools not well-suited for short peptides with smaller secondary structures. Consequently, we used the built-in structure similarity search in the RCSB PDB database, yielding numerous hits. Manual inspection and curation revealed that the majority of the structures achieved only limited alignment to pepitem on a global molecular scale; instead, many attained only fragment-wise acceptable scores. VhTI demonstrated the best conformational fit among the α-hairpinin structural family. The successful identification of the VhTI scaffold highlights the applicability of the implemented workflow, but to augment peptide scaffold selection, further improvements regarding the available algorithms for structure similarity searches are needed.

Structural scaffold matching can be enhanced by the integration of model predictions into the selection process prior to chemical synthesis and activity screening. In silico predictions of peptide macrocycles have undergone a huge boost with the release of the AF methods. For example, short macrocycles were predicted by deep neuronal network algorithms such as RoseTTAFold,^62^ also supporting peptide-drug conjugate designs, e.g., for the kappa opioid receptor.^63^ The AF methods, using a combination of deep learning and structural data sets, have progressed the field toward highly reliable predictions of natural peptides and proteins,^43^ and in particular for cyclic natural peptides with the AfCycDesign module.^34^ These methodologies are used to model huge peptide libraries, expanding on a large chemical space and diversity, to rapidly develop macrocyclic peptides for therapeutic targets.^64^ As proof-of-concept for the design of VhTI-pep 2, its structure was determined using NMR spectroscopy. The alignment to the AF3 model was very good (RMSD = 1.07 Å) and overall matched the fragment displayed in the 14-3-3ζ parent protein. As a result of the molecular grafting work, a superior stability in human serum of >48 h for VhTI-pep 2 compared to pepitem with ∼3 h was detected, making VhTI an attractive natural peptide scaffold for future work. The computational selection of scaffold peptides with a high conformational fit compared to the bioactive molecule, exemplified in this study with the 14-3-3ζ macromolecule, enhances the probability for successful peptide engineering and molecular grafting. The methodology is broadly applicable with the computational techniques accessible, supporting a wide application in peptide and protein science and drug development.

The lack of detailed molecular information on pepitem’s pharmacophore impedes the rational design of drug candidates. Sequence-activity relationship analysis of pepitem did not yield conclusive evidence regarding the bioactive sequence within the molecule in the past.^26^ For example, analysis of the pepitem sequence in chordata species revealed that the primary sequence of the peptide is highly conserved, allowing hypotheses on a minimal active sequence within the molecule. The study identified the pepitem segment (QGAEL^5–9^) as evolutionarily primed, suggesting that the matured sequence is likely to contain amino acids that are detrimental to activity. VhTI-pep 2 allowed for conformational analysis of the bioactive sequence motif, which was not possible so far for the unstructured native pepitem. Interestingly, the analysis confirmed that the QGA^5–7^ sequence was located at the interface between the helical segment and the loop, whereas EL^8–9^ were located in the loop segment. However, measurements indicated that QGA^5–7^ was less ordered than the rest of the peptide and therefore might show some degree of flexibility, which could result in a change of conformation during binding to target proteins. This nourished the hypothesis that secondary structural elements in pepitem are needed to display full activity. In support, we also noted that AF3 provided some prediction outputs of native pepitem with a short helical segment spanning the QGA^5–7^ interface (data not shown). On the sequence-activity relationship, Glu^4^ seemed to be of lower relevance for activity, as VhTI-pep 3 and 5 were poor probes, whereas a minor contribution to activity could be associated with Ser^10^, because VhTI-pep 1 was active as well. Inspection of the side chain orientation in the modeled structures of the grafted peptides, for example, the active VhTI-pep 2 was overall very similar to VhTI-pep 3. Interestingly, besides the additional Glu^9^ residue, only Gln^10^ was slightly changed in its spatial orientation compared to VhTI-pep 2 (Figure 3C), suggesting the Gln important for activity. The different analogs mapped conformational space, which was key for obtaining functional and bioactive peptides. At the end, only mimicking the one conformation present in the 14-3-3ζ protein crystal structure, which was the unbound state and might be significantly different than in the target-bound state, would likely reduce the probability of success of scaffold matching. Our results corroborate the recently proposed multiple pharmacophore model within pepitem,^26^ where at least two short pharmacophore sequences, SVT^1–3^ and QGA^5–7^, were reported.^22,26^ However, this also implies that more research is required to gain deeper insights into the pharmacophore chemistry of pepitem.

Taking advantage of the well-structured VhTI-pep 2 molecule, we modeled the peptide–protein interaction with the target proteins using AF3 multimer and AF3 for local interaction scoring. Interestingly, VhTI-pep 2 was predicted to interact with the Ig5 domain of Ncam-1, and the proposed interaction sites were in close proximity to the domain interface of the FN-1 domain and its adjacent Ig5 fragment. By comparison, linear pepitem was modeled to interact with the FN2 domain of the protein, similar to what was proposed previously.^35^ Several peptides targeting Ncam-1 Ig-like domains have been reported with agonistic or antagonistic pharmacology so far, with the Ig-like domains as ligand binding sites. Among them is the C3d peptide reported to initiate Ncam-1 dependent activation of signal cascades leading to functional effects such as neurite outgrowth.^46^ In silico interaction prediction of molecular contacts involving short linear peptide ligands is often poor compared to the prediction of protein–protein interactions.^66^ Therefore, hypothesis-generating modeling results should be validated by direct binding assays to wild-type or mutant protein, or similar experiments, to deliver independent confidence. Recent studies highlight improvements to model peptide–protein interactions, and enhanced workflows are being generated to improve the quality of the models to further support in silico evaluation of peptide–protein binding experiments in AF.^67,68^ It can be speculated that the well-structured VhTI-pep 2 allows for higher confidence in the prediction of interaction sites compared to the flexible pepitem.^69^ The stabilized VhTI-pep 2 can be considered a novel tool to facilitate experimental and in silico approaches. However, to confirm the actual binding site and binding interactions of the peptide, future studies need to experimentally validate these proposed binding sites. This study presents the first accurate structure of a pepitem pharmacophore, opening avenues for more SAR work as well as rational designs to promote future drug development.

Pharmacologic control of immune cell migration is a highly valued, clinically accepted therapy concept for the treatment of inflammatory and autoimmune disorders such as MS.^8,70^ For instance, the field is dominated by S1PR modulators of the fingolimod type. Pepitem has gained interest as a novel modulator of lymphocyte migration by effects on the crosstalk between the endothelium and lymphocytes. The therapeutic scope of the linear pepitem has been extensively evaluated in different models of inflammation and autoimmune disorders.^22–25^ Here we demonstrated, similar to what was previously described,^22^ that pepitem as well as VhTI-pep 2 treatment reduced the migration of T-cells with a maximal effect of up to 40% inhibition in vitro, and these effects were evident on different T-cell populations, including memory cells. Furthermore, VhTI-pep 2 inhibited the migration of memory and Th1 lymphocyte populations derived from blood samples of MS patients. This observation of partial inhibition of immune cell migration is based on the rather variable expression profiles of S1PR on immune cells, e.g., constitutive expression on naïve lymphocytes, T-cell activation reduces S1PR surface expression,^71,72^ or recirculating T-cells have higher S1PR expression compared to tissue resident cells.^73,74^ In particular, naïve T-cells are characterized by a constant S1PR expression,^13,75^ which makes them susceptible to peptide treatment. Additionally, the different memory phenotype T-cells show subtype-specific S1PR expression profiles.^15,73,74^ Central memory T-cells (TCM) have high S1PR expression, whereas effector memory cells express lower S1PR levels.^76^ Besides the effect on immune cell migration, studies suggested further immunomodulatory mechanisms of pepitem by changing cytokine production, e.g., TNF-alpha, IL-6, and IL-10, but the molecular mechanism beyond it remains unclear.^26^ Recently, CD56 has been identified as an additional target protein for pepitem, showing that CD56 activation by pepitem in osteoblasts leads to bone formation.^35^ Interestingly, CD56 is a known phenotype marker of human natural killer (NK) cells, where the protein is associated with cell function and trafficking.^77^ There is limited knowledge on the function of CD56 in other immune cells,^67^ but mechanisms beyond it may render pepitem immunomodulatory properties. Taken together, the observed effects on T-lymphocyte migration provide important evidence for the promising bioactivity of VhTI-pep 2, supporting its use as a tool in molecular pharmacology. VhTI-pep 2 can easily be functionalized at the free N-terminus, including PEGylation or lipidation to enhance plasma serum binding, as well as fluorophores to support fluorescence-based studies. Importantly, the enhanced metabolic stability supports therapeutic applications in inflammatory and autoimmune disease models.

At a more general level, the presented approach for a structure-based grafting strategy that combines structure-similarity identification of suitable scaffold peptides and computational modeling can be broadly applied to design a wide range of stabilized peptides (Figure 7). Overall, the methodology offers a rational procedure for scaffold selection and peptide design to improve the likelihood of successful grafting studies. The method is universal and applicable to similar bioactive peptides and may enhance the utilization of natural peptides as stabilizing peptide scaffolds for peptide engineering work in the future. The findings underscore the importance of structure-based grafting strategies in enhancing the efficiency and precision of molecular grafting workflows.

**Figure 7:**
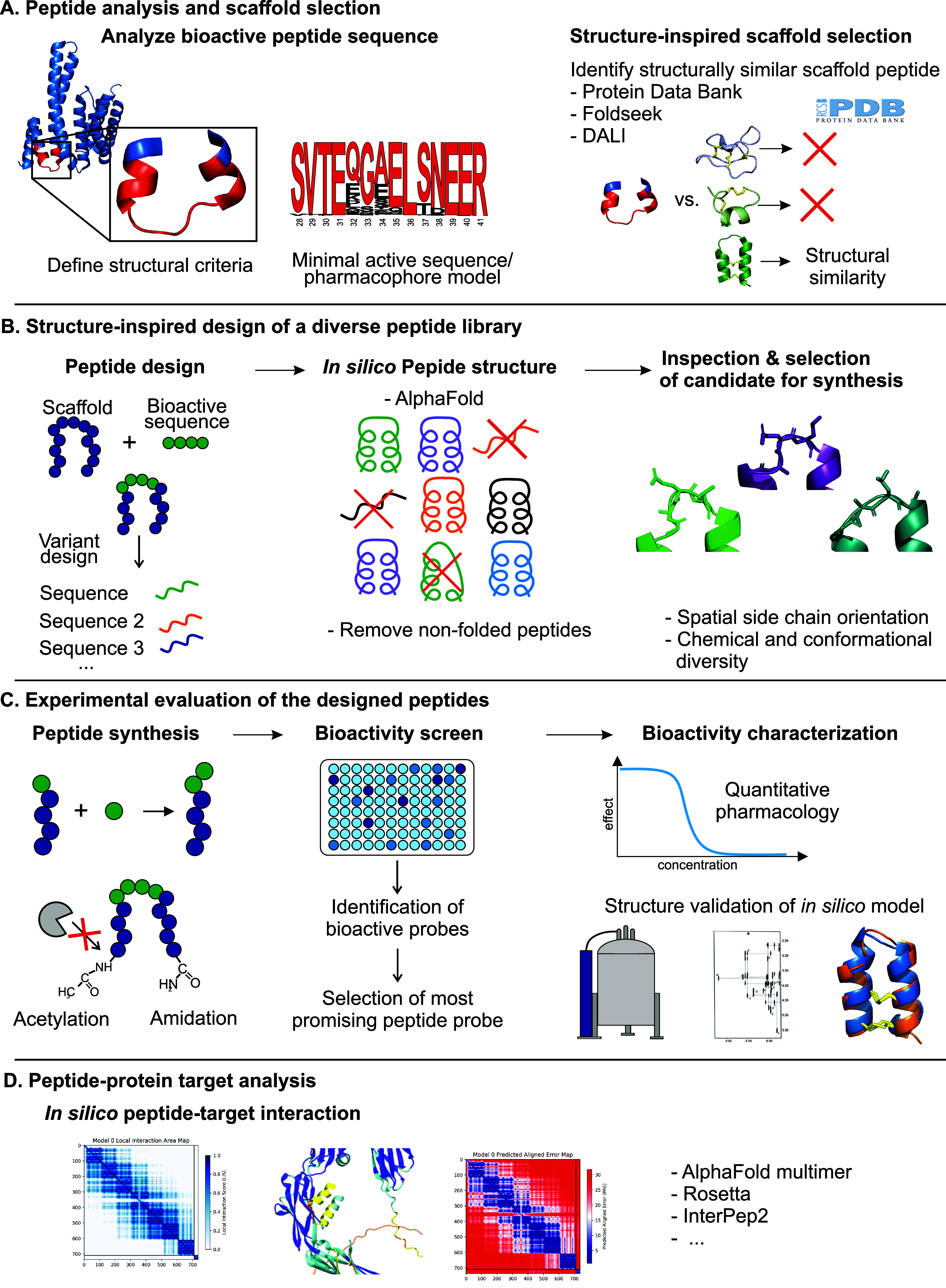
**Workflow for structure-scaffold matching and structure-inspired molecular grafting**. (A) Structure and conformation are crucially linked to the bioactivity and function of peptides, making information on structural motifs essential for molecular grafting studies. Conformational data can be obtained from experimentally resolved structures stored in publicly available databases. Predicted models, for example, can be utilized as well. In addition, defining the bioactive pharmacophore or the minimal bioactive sequence is a key step in selecting a peptide sequence, which can be integrated into a scaffold peptide. This can be achieved, for example, by analyzing evolutionary sequence conservation, performing alanine scanning, or testing truncated peptide sequences for bioactivity. Structure similarity searches are suitable to identify structurally analogous scaffold peptides, Foldseek, Dali, or the PDB’s search function supported this work. A curation of the output is needed based on predefined requirements, such as the presence of disulfide bonds, cyclization, or scaffold size. The obtained hit list is curated based on rational considerations regarding similarity to the bioactive sequence conformation. For example, calculating the RMSD values of the alignment of the scaffold with the bioactive peptide helps identify the scaffold peptide with the closest conformational fit to the target structure. (B) For the design of peptide libraries, secondary structures should be carefully considered. A diverse library of peptide sequences can be designed in this step. The conformation and folding of the designed peptides can then be assessed using in silico modeling tools such as AF. The predicted structures are manually inspected to filter out peptides that fail to adopt the native conformation properly. Further refinements of the library can be implemented to obtain a conformational and chemical diversity of the library. (C) In the chemical synthesis, the chemical toolbox is utilized to tailor the peptide, e.g., to increase the stability of the peptides. The synthesized peptide library is used for in vitro bioactivity screenings to identify bioactive peptide candidates. Hit peptide(s) undergo further pharmacological characterization, including measuring in vitro efficacy, potency, or further used for in vivo evaluations. Experimentally determined peptide structures may be confirmatory of the modeled peptide structure and serve as the basis for structure-based designs. (D) A structured peptide is suitable for homology modeling to predict binding interactions with target proteins using in silico methods, including tools like AF multimer. These experiments, which can offer insight into the peptide-target interaction, aid rational design of peptides. Moreover, peptides can be utilized for target identification through affinity-based pull-down assays or further mechanistic investigations of the signaling pathways.

## 4 Experimental Section

### 4.1 Chemicals

Acetonitrile (ACN), ammonium bicarbonate, dichloromethane (DCM), diethyl ether, Evans Blue, hydrochloric acid (HCl), methanol (MeOH), phenol, pyridine, trifluoroacetic acid (TFA), triisopropylsilane (TIPS), urea and α-cyano-4-hydroxycinnamic acid were purchased from Sigma-Aldrich (St. Louis, United States). Acetic acid and sodium dodecyl sulfate (SDS) were purchased from Carl Roth (Karlsruhe, Germany). Tris­(hydroxymethyl)­aminomethan (Tris) was purchased from Serva Electrophoresis GmbH (Heidelberg, Germany). Protease inhibitor tablets were purchased from Thermo Fisher Scientific (Waltham, USA). Sphingosine-1-phosphate was purchased from Cayman Chemical (Ann Arbor, United States). Lifitegrast was purchased from MedChemExpress (*South Brunswick, United States*).

### 4.2 Peptide Synthesis and Folding

Peptides were synthesized on an automatic Liberty Blue microwave peptide synthesizer (CEM Corporation, NC, USA).^35^ The VhTI–pepitem grafts were synthesized as C-terminal amides using Rink amide resin. Fmoc-AA (5 equiv) with suitable protecting groups was used, and to obtain defined folding, two of the cysteines were acetamidomethyl (Acm) protected. Fmoc deprotection was performed using 20% piperidine/DMF. Couplings were carried out with DIC/Oxyma-pure at 90 °C. The ratio of DIC/Oxyma-pure was 1:2. Upon completion of the peptide chain, the resin was washed with DCM/MeOH. After the seventh amino acid, double couplings were performed. After peptide assembly, indicated peptides were N-terminally acetylated under constant shaking for 20 min at room temperature (RT) in acetyl anhydride:pyridine:DCM (v/v/v, 1:1:4), after which the peptides were washed twice with DCM. Peptides were cleaved from the resin by treatment with Cleavage Cocktail B [TFA (88% v/v), phenol (5% v/v), water (5% v/v), and TIPS (2% v/v)] for 2 h, followed by diethyl ether precipitation. Cystine network peptides were synthesized with orthogonal Cys protecting groups, Acm-protected Cys (II and III) or *tert*-butyl-protected Cys (I and IV), by which a pair of cysteine residues can be deprotected and oxidized next to another. Oxidative folding was performed to establish the first disulfide bond.^36^ Peptides were dissolved at a concentration of 0.5 mg/mL in 0.1 M NH_4_HCO_3_, at pH 7.8, and folded at RT (25 °C) under constant stirring for 36 h. The intermediate peptides were purified by HPLC. To form the second disulfide bond, the peptides were dissolved at 4 mg/mL in acetic acid and 0.1 M HCl. 50 equiv. iodine (I_2_) per Acm group dissolved in MeOH were added to the peptide to reach a final dilution of acetic acid:0.1 M HCl:MeOH, v/v/v, 80:15:5. The mixture was incubated for 4 min at RT, after which the iodine was extracted with DCM from the aqueous phase and the fully folded peptides were isolated using preparative HPLC. In addition to synthesis with orthogonal Cys protection, VhTI-pep 2 was additionally synthesized with *tert*-butyl-protected Cys only and randomly folded correctly (data not shown) in 0.1 M NH_4_HCO_3_ (0.5 mg/mL peptide, pH 7.8 at room temperature for 36 h). The mass and peptide purity were confirmed by using MALDI-TOF MS and HPLC-UV analysis. All peptides used in this study were of ≥ 95% purity as determined by HPLC analysis with absorbance detection at 214 or 280 nm. The probe truncated pep-2 had ≥ 91% purity. The cystine connectivity of VhTI-pep 2 was confirmed by NMR spectroscopy.

### 4.3 High-Performance Liquid Chromatography

All HPLC experiments were performed in solvent A (0.1% TFA in ddH_2_O) and solvent B (90% ACN/10% ddH_2_0/0.1% TFA; v/v/v) using linear or isocratic (for peptide purification) gradients of solvent B. For analytical HPLC, a C_18_ column (150 mm × 3.0 mm, 2.6 μm, 100 Å, Kinetex Phenomenex) was used at a flow rate of 0.4 mL per min. Purification of synthesized peptides was performed on preparative and semipreparative C_18_ columns (250 × 21.2 mm, 10 μm, 300 Å Phenomenex Jupiter or 250 × 10 mm, 5 μm, 100 Å, Kromasil). Eluted peptides were observed using UV detection at 214 nm.

### 4.4 Mass Spectrometry

MALDI-TOF mass spectrometry was performed using an Autoflex Speed TOF/TOF MALDI-MS system (Bruker Daltonics, Bremen, Germany). Samples were mixed with a saturated α-cyano-4-hydroxy-cinnamic acid solution in ACN/ddH_2_O/TFA 50/50/0.1 (v/v/v) in a ratio of 1:6 and 0.5 μL was spotted on a ground steel MALDI target plate. After air-drying the spots, the mass spectra were acquired, and the obtained mass spectra were analyzed using FlexAnalysis software (Bruker Daltonics). The mass spectrometer was calibrated daily with Peptide Mix 4 (Laser Biolabs, Valbonne, France).

### 4.5 Cell Culture

Human microvascular endothelial cells (HMEC-1 cells, Cytion, Eppelheim, Germany) were cultured in MCDB-131 medium (with 2.2 g/L sodium bicarbonate, without l-glutamine, PAN-Biotech Aidenbach, Germany) supplemented with 10% fetal bovine serum (FBS, Capricorn Scientific, Ebsdorfergrund, Germany), 100 units/ml penicillin-streptomycin (Sigma-Aldrich) 10 ng/mL epidermal growth factor (BioLegend, San Diego, US), 1 μg/mL hydrocortisone (Sigma-Aldrich), and 10 mM l-glutamine (Sigma-Aldrich). For experiments, the cells were used between passage numbers 4–9. For culturing cells on transwell inserts, the cells were seeded on 0.1% gelatin (Sigma-Aldrich) coated polyethylenterephthalate (PET) transwell inserts with a pore size of 3 μm (Cellquart, Northeim, Germany). The cells were cultured in 150 μL medium in the upper side of the insert, and the lower compartment was filled with 600 μL cell culture medium. Peripheral blood mononuclear cells (PBMC) were isolated from the blood of healthy donors using lymphocyte separation medium (Capricorn Scientific). For this, venous blood was collected in K_2_EDTA blood collection tubes (Becton Dickinson, Plymouth, United Kingdom), diluted 1:1 in phosphate-buffered saline (PBS), and layered onto the lymphocyte separation medium, followed by centrifugation at 550×*g* for 30 min. The PBMC layer was transferred into a fresh tube and cells washed 3 times with PBS and either used directly or cultured in RPMI 1640 (Sigma-Aldrich) supplemented with 10% FCS and 100 units/mL penicillin-streptomycin. For migration assays with MS patient-derived PBMC, the PBMC were received as frozen stocks and were thawed, cultured overnight in medium, and used for migration assays on the next day.

### 4.6 Transepithelial-Transendothelial Electric Resistance Measurement (TEER)

Transepithelial transendothelial resistance measurement (TEER) of EC monolayers in the transwell inserts was measured using the Millicell ERS-2 (Merck Millipore, Burlington, USA) system with chopstick electrodes in resistance mode. Electrical resistance (Ω) was measured at the indicated time points after seeding EC into the transwell inserts. TEER values for cell monolayers were calculated as follows: TEER [Ω cm^2^] = (Ω cell monolayers – Ω empty ‘control’ insert)* surface area of the insert cm^2^; where Ω is the measured resistance.

### 4.7 Permeability Measurement

For permeability measurements, 0.67 mg/mL Evans Blue solution was prepared in 4% BSA in Hanks Buffered Salt Solution (HBSS, Thermo Fisher Scientific). Permeability through the cell monolayer was measured by the addition of 150 μL Evans Blue-labeled bovine serum albumin (BSA, Capricorn Scientific) solution in the upper compartment of the inserts on top of the EC and 600 μL of 4% BSA solution in HBSS into the lower compartment. The inserts were incubated at 37 °C for 4 h, after which samples from the lower compartment were collected and the absorbance measured at 620 nm. Concentrations (*C*) were calculated from a standard curve with known concentrations. The apparent permeability coefficient (*P*_app_) was calculated as *P*_app_ (cm/s) = (*C*_abluminal_ * Volume_abluminal_)/(insert surface area * *C*_luminal_ * time in seconds).

### 4.8 Quantitative Real-Time PCR

HMEC-1 cells were incubated for 16–20 h in cell culture medium with or without stimulation using 100 ng/mL tumor necrosis factor alpha (TNF-α, BioLegend) and 20 ng/mL interferon gamma (IFN-γ, BioLegend). RNA of the cells was purified using a Quick-RNA mini-prep RNA isolation Kit (Zymo Research) according to the manufacturer’s guidelines. 1,000 ng of isolated RNA were utilized for cDNA synthesis using the NxGen M-MuLV Reverse Transcriptase (Lucigen, Middleton, USA) with random hexamers (Carl Roth) and dNTP mixture (Thermo Fisher Scientific). Real-time PCR to quantify mRNA levels was performed on a CFX Connect Real-Time PCR Detection System (Bio-Rad, Hercules, USA), using the SsoAdvanced Universal SYBR Green Supermix (Bio-Rad) with 0.75 nM of each forward and reverse primer (Sigma-Aldrich) in a total of 10 μL reaction volume. Amplification was performed for 40 cycles of 10 s at 95 °C followed by 30 s at 60 °C. Gene levels were normalized to actin and described as fold increase in expression relative to unstimulated HMEC-1 cells. The used primer sequences are shown in Table S1.

### 4.9 Western Blot

Whole cell lysates were produced by lysis of cells in lysis buffer (6 M urea, 0.1% SDS with protease inhibitors) and insoluble material was removed by centrifugation at 16,000×*g* for 30 min. The protein content of the lysate was measured using the BCA protein assay (Thermo Fisher Scientific) according to the manufacturer’s protocol. Lysates were boiled in Laemmli buffer for 5 min at 95 °C. Ten μg of protein was loaded and separated using SDS-PAGE (5% stacking and 10% separation gel). Subsequently, the proteins were transferred onto a nitrocellulose membrane at 25 V for 30 min using a Trans-Blot Turbo System (Bio-Rad, Hercules, USA). Membranes were blocked with blocking solution (5% milk powder (Santa Cruz Biotechnology, Dallas, USA) in TBST buffer (20 mM Tris, 150 mM NaCl, 0.1% Tween, pH 7.6)) for at least 2 h followed by overnight incubation with primary antibodies, VCAM-1 (clone: A16047A, lot: B238253, Biolegend) and β-actin (clone: AC-15, lot: FAK20345–02, CarlRoth). To visualize the bands, membranes were incubated with HRP-conjugated secondary antibody (clone: Poly4053, lot: B329856 Biolegend) at RT for 2 h after which the bands were visualized using luminol reagent (Santa Cruz Biotechnology) on a FluorChem HD2 Imager (Alpha Innotech). Fiji (version 1.54f)^37^ was used to quantify protein levels.

### 4.10 Transwell Migration Assay

Transwell migration assays were performed similarly as published previously.^38^ HMEC-1 cells were seeded at a density of 75,000 cells per well in transwell inserts. Three days after cell seeding, the medium was changed, and the cells were stimulated with 100 ng/mL TNF-α and 20 ng/mL IFN-γ for 16–18 h. On the day of the assay, cells were washed with migration medium (serum-free RPMI medium supplemented with 0.1% BSA) to remove residual cytokines and starved for at least 1 h at 37 °C in migration medium. PBMC were starved for at least 1.5 h in migration medium before addition to the EC. For S1P and lifitegrast-treated samples, the PBMC were preincubated for at least 1.5 h at 37 °C with the ligands before addition to the EC. Pepitem and other peptides were added onto the EC monolayer and preincubated for 15–30 min at 37 °C before the addition of the PBMC. 200,000 PBMC per well were added directly on top of the EC prestimulated with pepitem or other peptides to reach a final volume of 150 μL in the donor compartments. The receiver compartments of the wells were filled with 600 μL RPMI with 20% FBS. After 4 h of migration at 37 °C, the receiver compartment with the migrated cells was collected. The migrated cells were stained with the indicated antibodies: CD3-APC (clone: OKT3, lot: B400127), CD4-PE (clone: OKT4. lot: B379180), CD8-FITC (clone: HIT8a, lot: B370193) from Biolegend and CD3-VioGreen (clone: REA613, lot: 5240502518), CD45RO-FITC (clone: REA611, lot: 5240502519), CD4-PerCP-Vio700 (clone: REA623, lot: 5240506702), CXCR3 PE-Vio615 (clone: REA232, lot: 5240506722), CD196-APC (clone: REA190, lot: 5240506717), CD8-APC-Vio770 (clone: REA734, lot: 5240303993) and CD45RO-PEVio616 (clone: REA611, lot: 5231102514) from Miltenyi Biotec (Bergisch Gladbach, Germany) and counted using precision counting beads (Biolegend) on a CytoflexS flow cytometer (BeckmanCoulter). Data was analyzed using CytExpert (Version 2.4). T cells were gated as CD3^+^ cells, T cell subsets were gated as CD4^+^ (CD3^+^, CD4^+^), CD8^+^ (CD3^+^, CD8^+^), memory CD4^+^ (CD3^+^, CD4^+^, CD45RO^+^), memory CD8^+^ (CD3^+^, CD8^+^, CD45RO^+^) and Th1 cells (CD3^+^, CD4^+^, CXCR3^+^, CCR6^–^).

### 4.11 Serum Stability Assay

Stability of peptides in human serum was measured similarly to that previously described.^34^ In short, fresh blood from healthy donors was collected in serum collection tubes (Clot Activator Tube, Becton Dickinson, Plymouth, United Kingdom, with anticoagulant) and incubated at 4 °C for 2 h to allow blood clotting. The serum was separated by centrifugation at 1000 × *g* for 10 min. To remove lipids from the serum, an additional centrifugation step was performed at 15,000 × *g* for 15 min at 4 °C, after which the serum was used for the stability assays. For this, the serum was prewarmed for 10 min at 37 °C before the addition of the test compounds. Peptides were prepared at a 10-fold concentration in PBS and diluted to a final concentration between 60 and 30 μM with the serum. The serum-peptide reaction mix was incubated at 37 °C, and 40 μL aliquots were taken at the indicated time points. To stop the reaction, samples were mixed with 40 μL of 6 M urea for 10 min on ice, followed by precipitation of serum proteins by the addition of 40 μL of 20% TFA and incubation for at least 10 min on ice. The precipitate was pelleted at 15,000 × *g* for 15 min, and 90 μL of the supernatant was freeze-dried. The dry material was resuspended in HPLC solvent A (0.1% TFA in ddH_2_O). Peptide quantification was performed using analytical HPLC. The remaining peptide relative to the starting point (time point 0) was calculated by peak integration to determine the area under the peak at the designated time points and further calculated in comparison to time point 0 min, which was set as 100%. To calculate the half-life of the peptides in human serum, data points were fitted with a one-phase decay equation.

### 4.12 Cell Cytotoxicity Assay

75,000 HMEC-1 cells per well were seeded in a 96-well plate and incubated for 3 days. On day 4, the medium was changed to 100 μL migration medium (0.1% BSA in serum-free RPMI medium). Cells were incubated with the test peptides for 4 h, after which 10 μL CCK8 reagent (TargetMol, Boston, USA) was added. Absorbance at 450 nm of the sample wells was measured after 2 h incubation with the CCK8 reagent using a Synergy H4 plate reader (Biotek, Winooski, USA). Incubation of cells with 150 μg/mL of the cytotoxic agent (S)-(+)-camptothecin (Glentham Life Sciences, Corsham, United Kingdom) was used as a positive control.

### 4.13 Sequence Analysis of 14-3-3 Proteins and PDB Database Search

Sequence homology of the pepitem sequence in 14-3-3ζ proteins among the taxon ID chordata was analyzed using the NCBI protein–protein blast tool (https://blast.ncbi.nlm.nih.gov/Blast.cgi).^40^ For this, the pepitem sequence (SVTEQGAELSNEER^1–15^) was used as the query sequence, the nonredundant protein sequences database was searched using the PAM30 matrix for scoring, and the search was restricted to the taxon ID chordata (taxid: 7711). Hits were manually curated to remove all sequences not belonging to the 14-3-3ζ protein family, and only one sequence per species was included. The WebLogo Tool^41^ was used to prepare the sequence logos. For searching the PDB database (https://www.rcsb.org/)^42^ for conformationally similar structures, the pepitem sequence structure (SVTEQGAELSNEER^28–41^) derived from PDB: 1QJB was used as the query sequence in the advanced search mode for structure similarity.^43^ The search was performed in the relaxed search mode, searching for chains with the results returned as structures. The obtained hits were further filtered for peptide and small protein entries, after which the hit list was downloaded and manually filtered for peptides with 10–50 amino acids with at least two Cys residues, and duplicate and redundant structures were removed before structural grouping of the results. Representative structures from each group were aligned to the pepitem structure in PyMOL, as described below. To allow alignment, an elongated structure derived from PDB: 1QJB (ACMKSVTEQGAELSNEERNLLS^24–45^) was utilized. Protein and peptide structures were visualized with PyMOL 2.5.5 (The PyMOL Molecular Graphics System, Version 2.5.5 Schrödinger, LLC.). RMSD values were calculated using the ‘align’, ‘super’ or ‘cealign’ command in PyMOL as indicated.

### 4.14 AlphaFold Modeling of Peptide Structures

Peptide structures were predicted using AF2 ^33^ together with a modified workflow for the improvement of confidence and accuracy of prediction of cyclic peptides.^34^ The disulfide bonds of the predicted AF structures of VhTI-pep 3 and VhTI-pep 6 were manually curated in PyMOL. We used AF3 ^44^ to generate 3D models of protein–protein complexes involving VhTI-pep 2, pepitem, CDH15, and CD56. Both full-length proteins and selected fragments were modeled to assess differences in predicted binding interactions. The highest confidence models obtained from AF3 were further refined. Missing hydrogen atoms and side chains were added using the Protein Preparation Wizard implemented in the Schrödinger Suite. The structure was optimized at pH 7.0 to ensure proper protonation states of ionizable residues. Subsequently, energy minimization was performed to relieve steric clashes and improve the overall geometry of the model. The minimization was carried out using the OPLS4 force field with a convergence threshold of 0.3 Å for heavy atoms. Model quality was evaluated using plDDT, a confidence metric for local structural regions, as well as the interface predicted TM-score (ipTM) and predicted TM-score (pTM) to evaluate interfacial and global structural accuracy, respectively. To analyze localized protein–protein interaction interfaces, we calculated the Local Interaction Score (LIS).^45^ Visualization of the 3D structures was performed using VMD (Visual Molecular Dynamics),^46^ with models color-coded by plDDT scores to highlight structural confidence. Interactions between Ncam-1 and VhTI-pep 2 were predicted using the protein–ligand interaction profiler online tool.^47^

### 4.15 NMR Spectroscopy and Structure Determination

For the structure determination of VhTI-pep 2 NMR spectra were recorded using a Bruker Avance III HD 700 MHz NMR spectrometer equipped with a cryoprobe. The peptide was prepared in 90% H_2_O/10% D_2_O, pH 5.1, at a concentration of ∼ 1 mg/mL. Sodium trimethylsilylpropanesulfonate (DSS) was added as an internal standard. A series of NMR experiments were collected at 298 K for this peptide, including: ^1^H, ^1^H TOCSY (80 ms mixing time), ^1^H, ^1^H NOESY (200 ms mixing time), ^1^H–^15^N HSQC, ^1^H–^13^C HSQC, and a ^1^H-TOCSY–^13^C-HSQC. Additional ^1^H, ^1^H TOCSY experiments were run at 288, 293, 298, 303, and 308 K to determine amide proton temperature dependence. Standard Bruker pulse sequences and excitation sculpting water suppression techniques were used for all experiments. Spectra were referenced to the DSS signal at 0.00 ppm and all spectra were processed using Topspin.

Following data acquisition, the NMR data were analyzed using the program Computer Aided Resonance Assignment (CARA).^48^ Resonance assignments were achieved using sequential assignment strategies, with the additional help of the ^13^C experiments recorded at natural abundance.^49,50^ ^1^H chemical shifts were subsequently used to assign the ^15^N data. Sample NMR data are presented in Figure S9. Structure calculations were initially performed in CYANA 3.98.^51^ Cross peaks in the NOESY data were integrated in CARA and automatically assigned and converted into distance restraints in CYANA. TALOS-N was employed to generate dihedral angle restraints by correlating chemical shifts with backbone and side chain torsion angles.^52^ Additional side chain dihedral restraints were derived from analysis of coupling constants and NOE patterns,^49^ as well as for cystine residues through analysis of chemical shifts using the program DISH.^53^ Finally, hydrogen bond restraints were derived from analysis of amide proton temperature coefficients and included for amide protons with a temperature coefficient of > −4.6 ppb/K if a suitable acceptor could be identified in the preliminary structure.^54^ The final structures were calculated and energy minimized in explicit water using the program Crystallography and NMR system (CNS).^55^ The force field building blocks for modified amino acids (norleucine at position 6 and acetylated glutamic acid at the N-terminus) were generated for both CYANA and CNS using the Automatic Topology Builder,^56^ as previously described.^57^ In the final round of calculations, 100 structures were generated, and the best 20 models chosen based on no violations from experimental data, low energies, and good stereochemical quality, as assessed by MolProbity.^58^

### 4.16 Data Analysis

Data were analyzed using GraphPad Prism software. EC_50_ values were calculated by three-parameter nonlinear regression analysis. For this, the data points were normalized to the number of migrated cells in untreated (buffer) control samples (100%), and the regression was fitted with the top constraint to 100 and the slope fixed to 1. Statistical significance is indicated in graphs as n.s. not significant, * *p* < 0.05; ** *p* < 0.01; *** *p* < 0.001. The statistical tests used are indicated in the respective figure captions.

## Supplementary Material







## Notes

Endothelial cell characterization and monolayer validation; quality control analysis of synthesized peptides; bioactivity of pepitem; sequence logos of the pepitem region of 14-3-3ζ proteins in chordata; quality control analysis and bioactivity of synthesized truncated pepitem variants; structure-inspired scaffold selection for peptide design; structural models of the peptide probes; serum stability and cytotoxicity of the peptide probes; structural analysis of VhTI-pep 2 using NMR; EC_50_ determination of VhTI-pep 2 for different T-cell subsets; peptide–protein interaction models for pepitem; peptide–protein interaction models between CDH15 and VhTI-pep 2; and Western blot membranes (PDF)
Pepitem sequence in chordata species and identified hit peptides in the PDB structure similarity search (XLSX)
Molecular strings formula spreadsheet of lifitegrast and S1P (CSV)

## Abbreviations

Acm: acetamidomethyl
ACN: acetonitrile
AF: AlphaFold
CDH15: cadherin 15
DCM: dichloromethane
EC: endothelial cell
FBS: fetal bovine serum
Fmoc: fluorenyl-methoxycarbonyl
HCl: hydrochloric acid
HMEC-1: human microvascular endothelial cells-1
ICAM-1: intracellular cell adhesion molecule-1
IFN-γ: interferon gamma
MeOH: methanol
MS: multiple sclerosis
NCAM-1: neural cell adhesion molecule
*P*_app_: apparent permeability coefficient
pepitem: peptide inhibitor of transendothelial migration
PBMC: peripheral blood mononuclear cells
RRMS: relapsing-remitting multiple sclerosis
SAR: structure–activity relationship
S1P: sphingosine-1-phosphate
S1PR: sphingosine-1-phosphate receptor
SDS: sodium dodecyl sulfate
SFTI-1: sunflower trypsin inhibitor-1
SphK1: sphingosine kinase 1
Spns2: spinster homologue 1
TEER: transepithelial transendothelial resistance
TEM: transendothelial migration
TFA: trifluoroacetic acid
TIPS: triisopropylsilane
TNF-α: tumor necrosis factor alpha
VCAM-1: vascular cell adhesion molecule 1
VhTI: *Veronica hederifolia* trypsin inhibitor
