# Supplementary material for: Engineering Peptide Modulators for T‑Cell Migration by Structural Scaffold Matching
Source: J Med Chem. 2025 Aug 12;68(16):17202–20. doi: 10.1021/acs.jmedchem.5c00677 (PMC12406255; doi:10.1021/acs.jmedchem.5c00677)
Supplement: Supplementary file 1 [file jm5c00677_si_001.pdf]

Supporting Information

Engineering Peptide Modulators for T-cell Migration by Structural Scaffold Matching

Jasmin Gattringer[a], Simon Hasinger[a], Agnes Weidmann[a], Katarzyna Walczewska-Szewc[b], Kirtikumar B. Jadhav[c], Tobias Zrzavy[d,e], Anja Steinmaurer[d,e], Paulien Baeten[f], Monika Perisic[c,g], Wilson Cochrane[h], Markus Muttenthaler[c,i], Bieke Broux[f], Dagmar Gotthardt[j], K. Johan Rosengren[h], Christian W. Gruber[a] and Roland Hellinger[a*]

[a] Center for Physiology and Pharmacology Medical University of Vienna, 1090 Vienna, Austria

[b] Institute of Physics, Faculty of Physics, Astronomy and Informatics, Nicolaus Copernicus University in Toruń, 87-100 Toruń, Poland

[c] Faculty of Chemistry, Institute of Biological Chemistry, University of Vienna, 1090 Vienna, Austria

[d] Comprehensive Center for Clinical Neurosciences and Mental Health, Medical University of Vienna, 1090 Vienna, Austria

[e] Department of Neurology, Medical University of Vienna, 1090 Vienna, Austria

[f] Department of Immunology and Infection Biomedical Research Institute, Hasselt University, 3590 Diepenbeek, Belgium

[g] Vienna Doctoral School in Chemistry, University of Vienna, 1090 Vienna, Austria

[h] School of Biomedical Sciences, The University of Queensland, Brisbane, Queensland 4072, Australia

[i] Institute for Molecular Bioscience, The University of Queensland, Brisbane, Queensland 4072, Australia.

[j] Department for Biological Sciences and Pathobiology, Pharmacology and Toxicology, University of Veterinary Medicine Vienna, 1210 Vienna, Austria

[#]Corresponding author: Roland Hellinger, Tel.: +43-(0)1-40160-31393, Email: roland.hellinger@meduniwien.ac.at

1 List of Supplementary Figures and Tables

Figure S1. Endothelial cell characterization and monolayer validation S3
Figure S2. Quality control analysis of synthesized peptides. S4
Figure S3. Bioactivity of pepitem. S5
Figure S4. Sequence logos of the pepitem region of 14-3-3ζ proteins in chordata. S6
Figure S5. Quality control analysis and bioactivity of synthesized truncated pepitem variants. S7
Figure S6. Structure-inspired scaffold selection for peptide design. S8
Figure S7. Structural models of the peptide probes. S9
Figure S8. Serum stability and cytotoxicity of the peptide probes. S10
Figure S9. Structural analysis of VhTI-pep 2 using NMR. S11
Figure S10. EC_{50} determination of VhTI-pep 2 for different T-cell subsets. S13
Figure S11. Peptide-protein interaction models between CDH15 and pepitem. S14
Figure S12. Peptide-protein interaction models between CDH15 and VhTI-pep 2. ...... S15
Figure S13. Western blot membranes. S16

Table S1. Primer sequences used for quantitative RT-PCR. S17
Table S2. Pepitem sequence in chordata species. S17
Table S3. Sequences of truncated pepitem probes and quality control of the synthesized peptides. S17
Table S4. Identified hit peptides in the PDB structure similarity search. S17
Table S5. RMSD calculation for alignment of the pepitem structure to scaffold peptides. S18
Table S6. Sequences of additionally modeled peptides. S19
Table S7. Alignment of predicted VhTI and VhTI-pep 1-6 structures. S19
Table S8. NMR structural statistics for VhTI-Pep2. ...... S20
Table S9. Proposed protein-protein interactions between NCAM-1 and VhTI-pep2 using the protein-ligand interaction profiler online tool. S21
Table S10. AlphaFold-Multimer prediction of interacting residues between VhTI-pep 2 and target proteins. S22

Figure S1. Endothelial cell characterization and monolayer validation. The model EC-line HMEC-1 was characterized for protein expression. To facilitate immune cell migration and model inflammatory conditions in vitro, EC were simulated with TNF-α and IFN-γ. This cytokine stimulation is known to induce the expression of adhesion proteins [1]. The expression of adhesion proteins **(A)** VCAM-1 and ICAM-1 was confirmed using qPCR and as expected, an increase in mRNA levels was measured in cytokine stimulated compared to unstimulated HMEC-1 cells. **(B)** Up-regulation of VCAM-1 expression upon cytokine treatment on the protein level was confirmed with Western blot analysis. **(C)** Sphingosine kinase 1 (Sphk1), spinster homolog 2 (Spns2) and cadherin 15 (CDH15) expression was confirmed in HMEC-1 cells using qPCR comparing cytokine-stimulated and unstimulated cells. **(D)** Monolayer formation of EC over 5 days in the transwell inserts was studied by measuring the transepithelial transendothelial electrical resistance (TEER) of different cell seeding densities, which reached a peak at 25.3 ± 2.2 Ωcm² at day 4 post-seeding of 75,000 cells/insert. **(E)** The apparent permeability (P_{app}) for 75,000 cells / well in the insert for Evans Blue-labeled bovine serum albumin was measured over the course of 5 days compared to empty transwell inserts yielding and baseline value of $1.1x10^{-6} \pm 1.7x10^{-6}$ cm²/s. **(F)** In the transwell migration assay, significantly more $CD3^{+}$ T-cells migrated through cytokine stimulated than through unstimulated EC monolayers. **(G)** Lifitegrast a known inhibitor of the lymphocyte function-associated antigen 1 integrin (LFA-1) inhibits migration of $CD3^{+}$ T-cells using the transwell assay with an EC_{50} of 2.2 ± 3.6 nM, similar as previously reported [2]. Data are shown as mean ± SD for 3 (A, B, C, D, F, G) or 4 **(E)** biological replicates. Significance was tested using student's t-tests. For qPCR experiments, statistical testing was performed on ΔCt values comparing stimulated and non-stimulated cells for each gene. Statistical significance is indicated in the graphs as: n.s. (not significant), * p<0.05; ** p< 0.01; *** p<0.001.

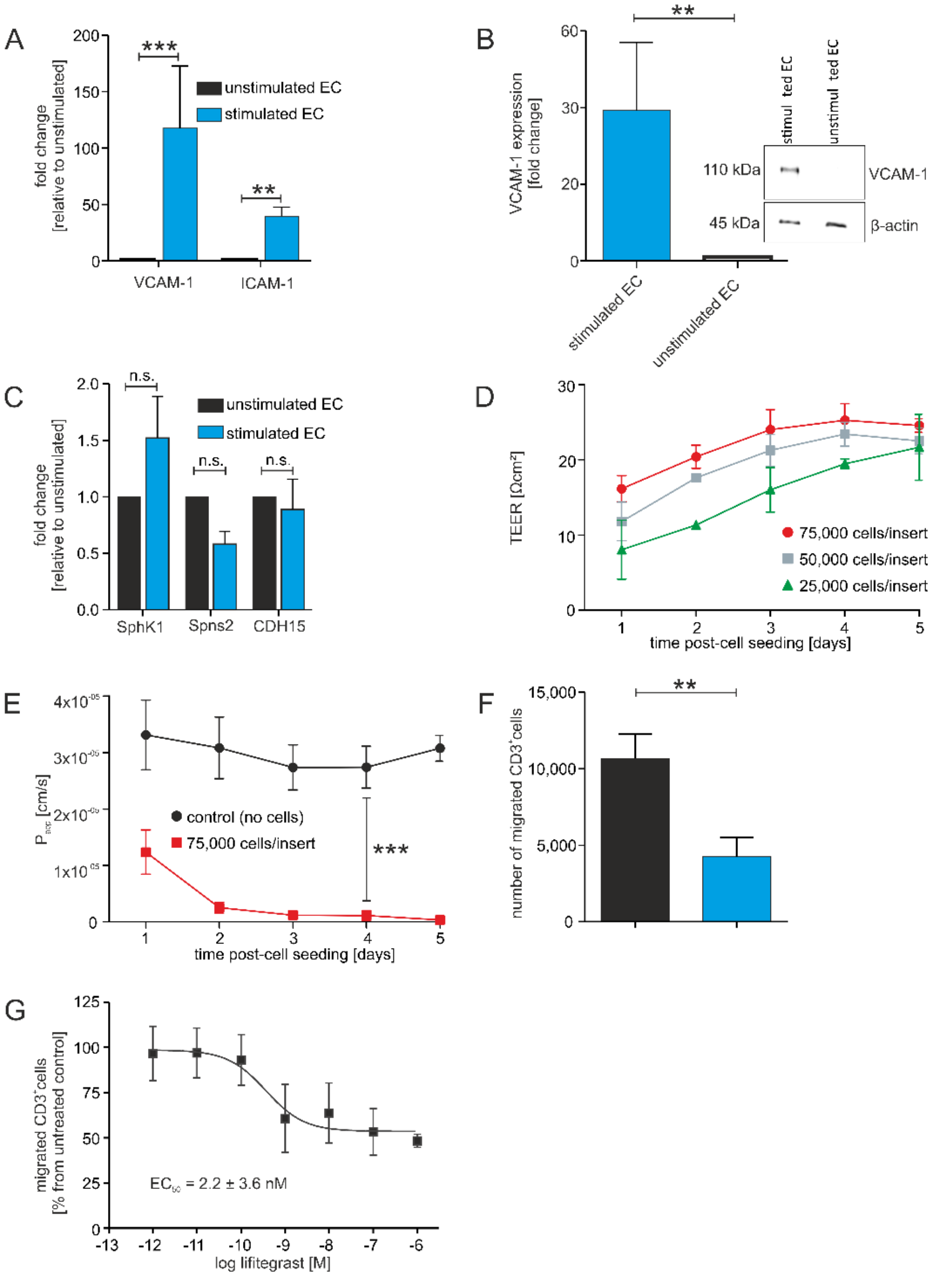

Figure S2. Quality control analysis of synthesized peptides. Analytical HPLC traces show the absorbance at 214 nm. The inserts provide the MALDI-TOF MS traces for each peptide and the monoisotopic peak $[M+H]^+$ is labeled. **(A)** pepitem, **(B)** VhTI-pep 1, **(C)** VhTI-pep 2 **(D)** VhTI-pep 3, **(E)** VhTI-pep 4, **(F)** VhTI-pep 5, **(G)** VhTI-pep 6 and **(H)** the VhTI scaffold.

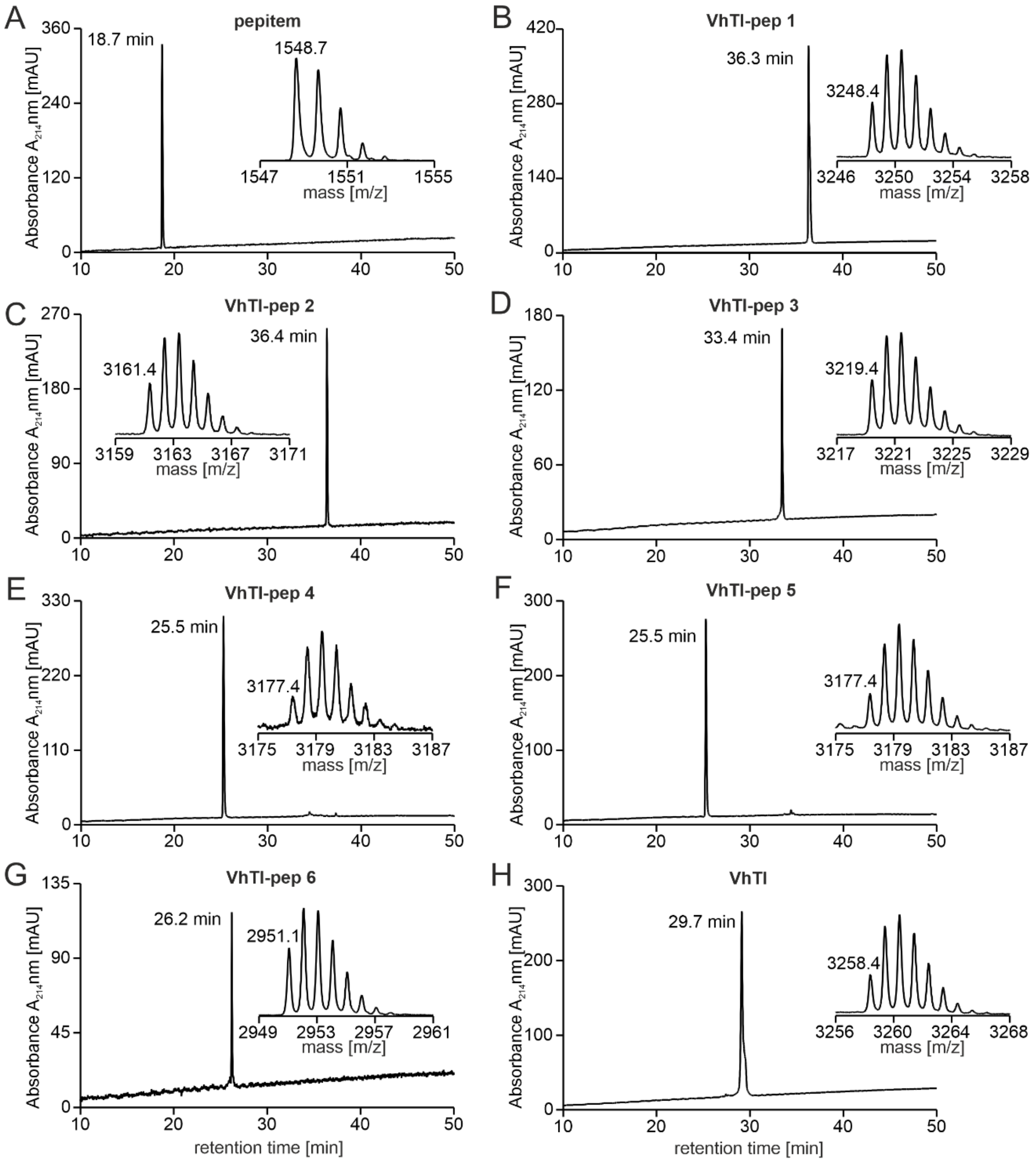

Figure S3. Bioactivity of pepitem. Pepitem was tested in a concentration-dependent manner for modulation of T-lymphocyte migration for **(A)** $CD4^+$ and $CD8^+$ populations as well as for **(B)** memory $CD4^+$ and $CD8^+$ cells. Data are shown as mean ± SD of 5 independent experiments.

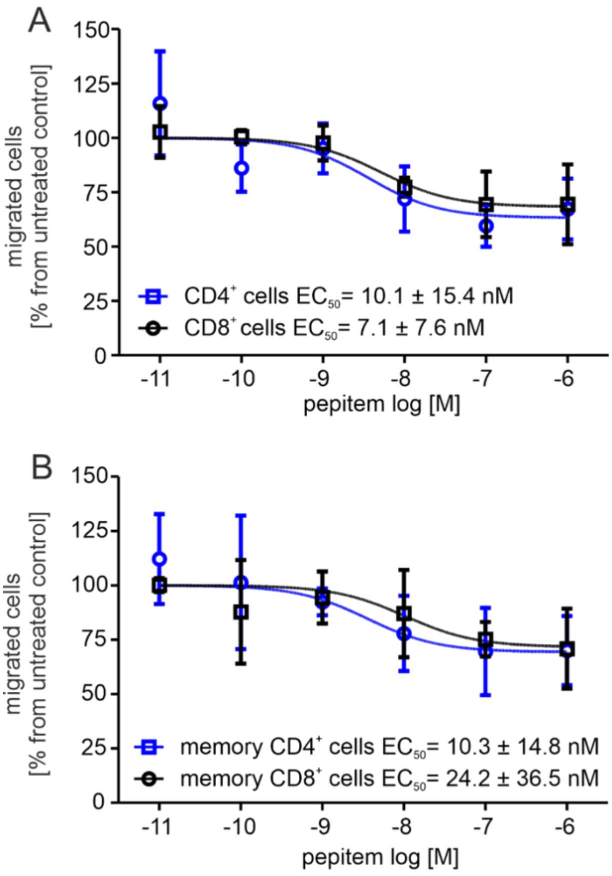

Figure S4. Sequence logos of the pepitem region of 14-3-3ζ proteins in chordata. A sequence similarity of 14-3-3ζ proteins was conducted in chordata species and of the 290 resulting species, subgroups were analyzed for **(A)** mammals (113 sequences), **(B)** fish (129 sequences), **(C)** birds (19 sequences), **(D**) amphibians (13 sequences) and **(E)** reptiles (16 sequences). The human pepitem sequence is highlighted in red, and other amino acids are shown in black. The amino acid residues are labeled based on the position in the human protein. The corresponding sequences are shown in Table S2.

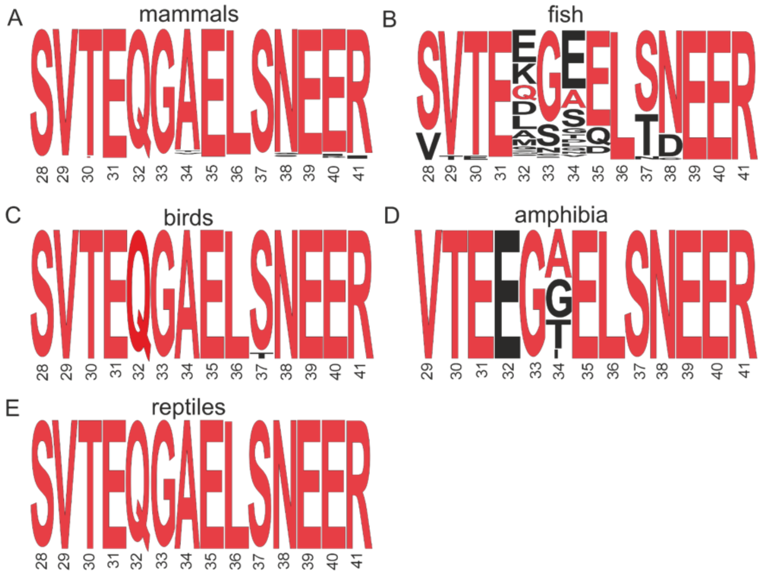

Figure S5. Quality control analysis and bioactivity of synthesized truncated pepitem variants. Analytical HPLC traces are shown for the absorbance at 214 nm. The inserts provide the MALDI-TOF MS traces for each peptide and the monoisotopic peak $[M+H]^+$ is labelled for **(A)** truncated pep-1, **(B)** truncated pep-2 and **(C)** truncated pep-3. **(D)** Bioactivity of the truncated variants in the transwell migration assay for inhibiting migration of $CD3^+$ cells was tested at 10 µM, 1 µM and 0.1 µM in comparison to the untreated control and the linear pepitem at 0.1 µM. Data are shown as mean ± SD for three independent measurements, statistical analysis was performed using repeated measures one-way ANOVA with Dunnett's post hoc test. Statistical significance is indicated in the graphs as: n.s. (not significant).

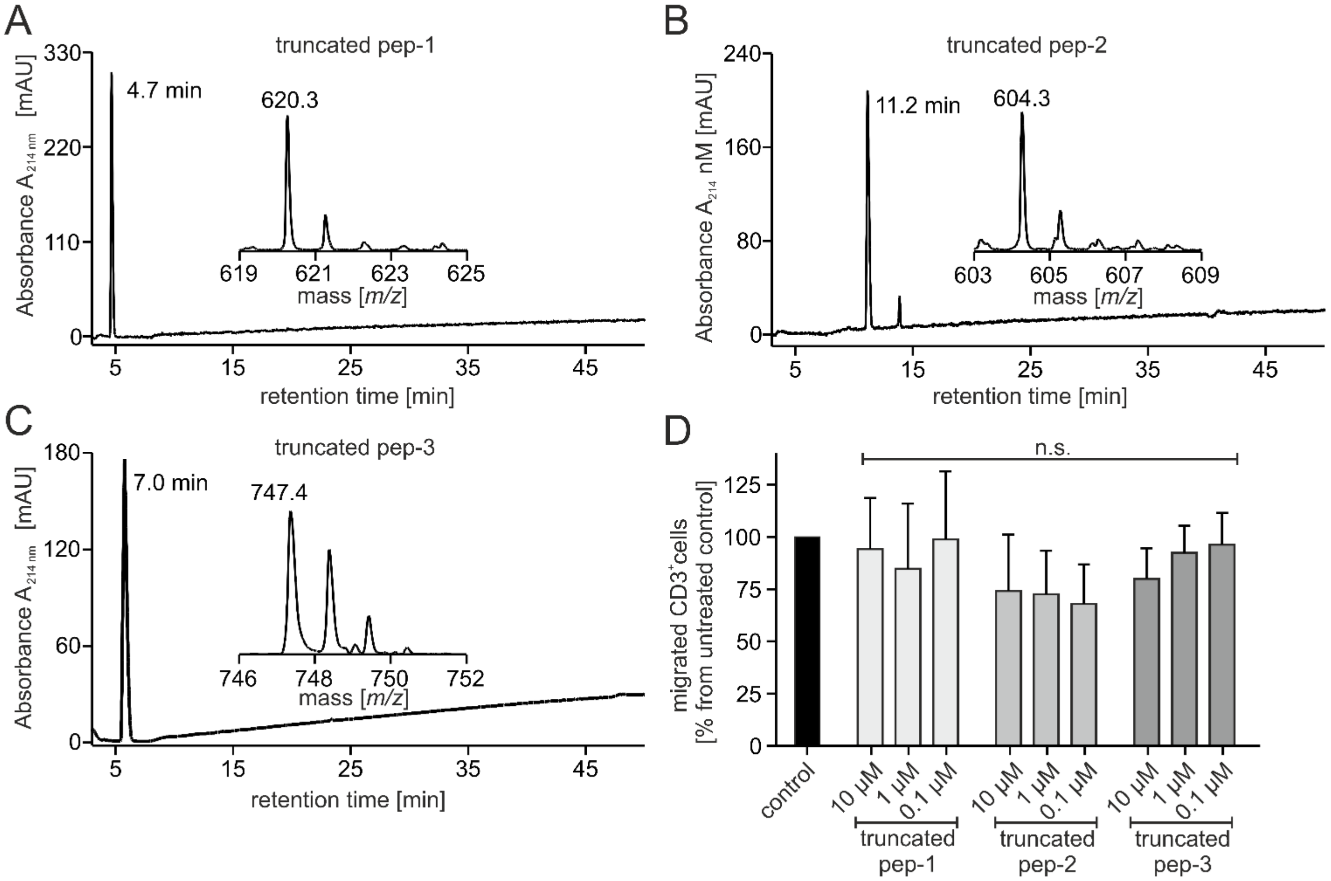

Figure S6. Structure-inspired scaffold selection for peptide design. (A) Grouping of the identified conformationally similar peptides to the pepitem structure. The 110 structures were grouped into: α-hairpinins, bowman-birk inhibitor peptides (BBI), β-hairpinins, conotoxins, endothelin-derived, enterotoxin-derived, knottins, scorpion toxins, other 1 disulfide bond network peptides, other two disulfide bond network peptides and others for structures which did not fit into the previously mentioned groups. All peptides identified in the search are presented in Figure S4. **(B)** Representative structures for each group were evaluated via alignment to the pepitem motif (derived from PDB: 1QJB): BBI (PDB: 1JBL), scorpion toxin (PDB: 1DU9), conotoxin (PDB: 1MVI), β-hairpinin (PDB: 1RKK), endothelin-derived (PDB: 1EDN), enterotoxin-derived (PDB: 1ETL), knottin (PDB: 1QFD), other one disulfide bond network peptides (PDB: 1PAO), other 2 disulfide bond network peptides (PDB: 1KWE) and others (PDB: 1T0W). In addition, the alignment to prototypic scaffold peptides (kalata B1 PDB: 1NB1, θ-defensin-1 PDB: 2LYF, and MCoTI-II PDB: 1IB9), which were not hits listed in the database search was performed as well. **(C)** Alignments to other members of the α-hairpinin peptide family are shown. The 14-3-3ζ derived structure used for alignment to the scaffold peptides is shown in transparent red, aligned to each structure. The alignments were performed using the “super” and “cealign” command in PyMOL. The calculated RMSD values as well as the used PDB entries are reported in Table S5.

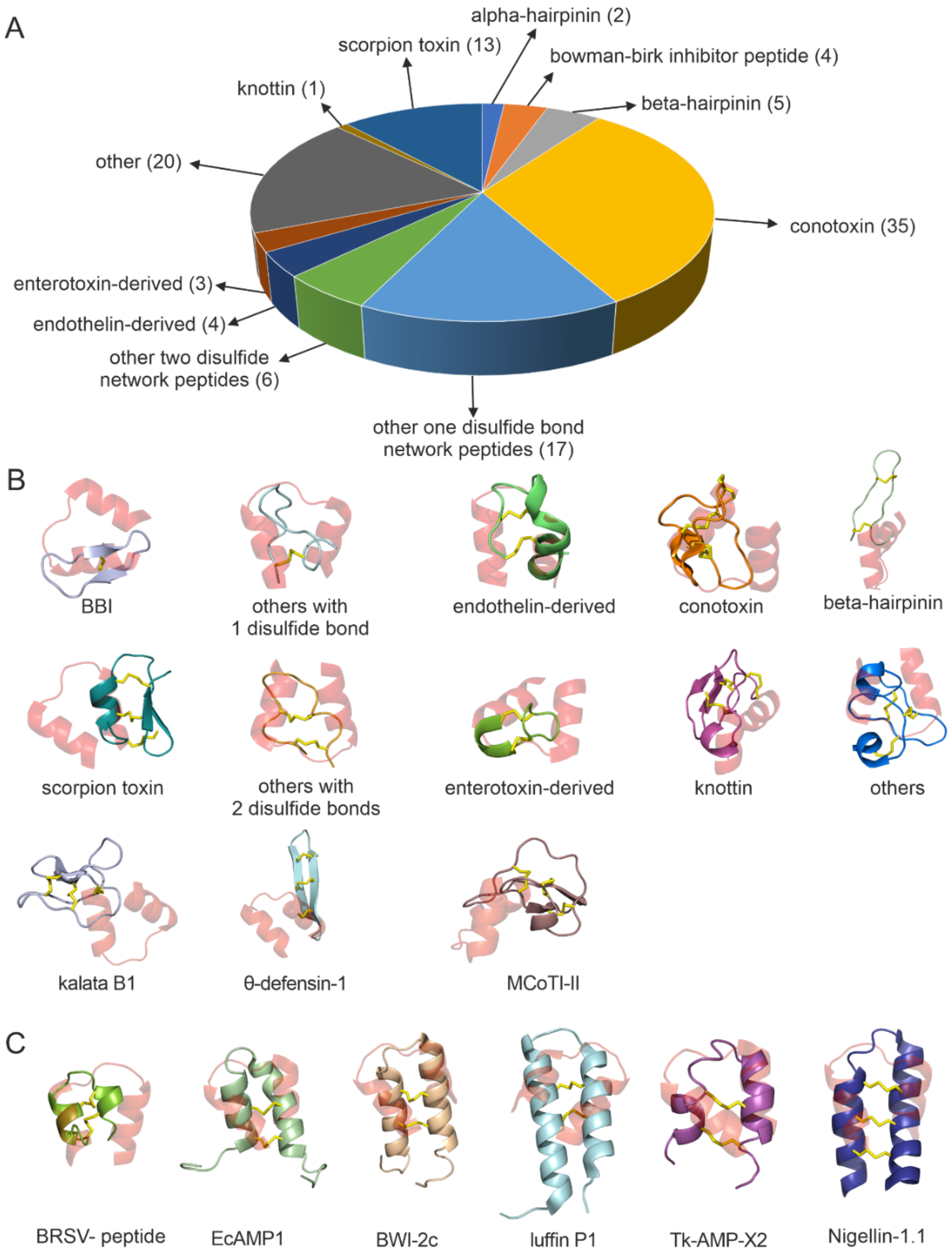

Figure S7. Structural models of the peptide probes. **(A)** The structures of the designed peptides were predicted using AlphaFold2, together with a workflow for modeling cyclic peptides [3, 4]. The VhTI scaffold structure (truncated VhTI) was derived from published data (PDB: 2PLX); the structure labeled as VhTI shows the modeled structure of the peptide using AlphaFold2. Disulfide bonds are shown in yellow. **(B)** Quality control for the predicted peptide models showing the predicted distance difference test (left graph) and the predicted aligned error for every residue in the peptides (right graph) for VhTI-pep 1 to 6 and VhTI.

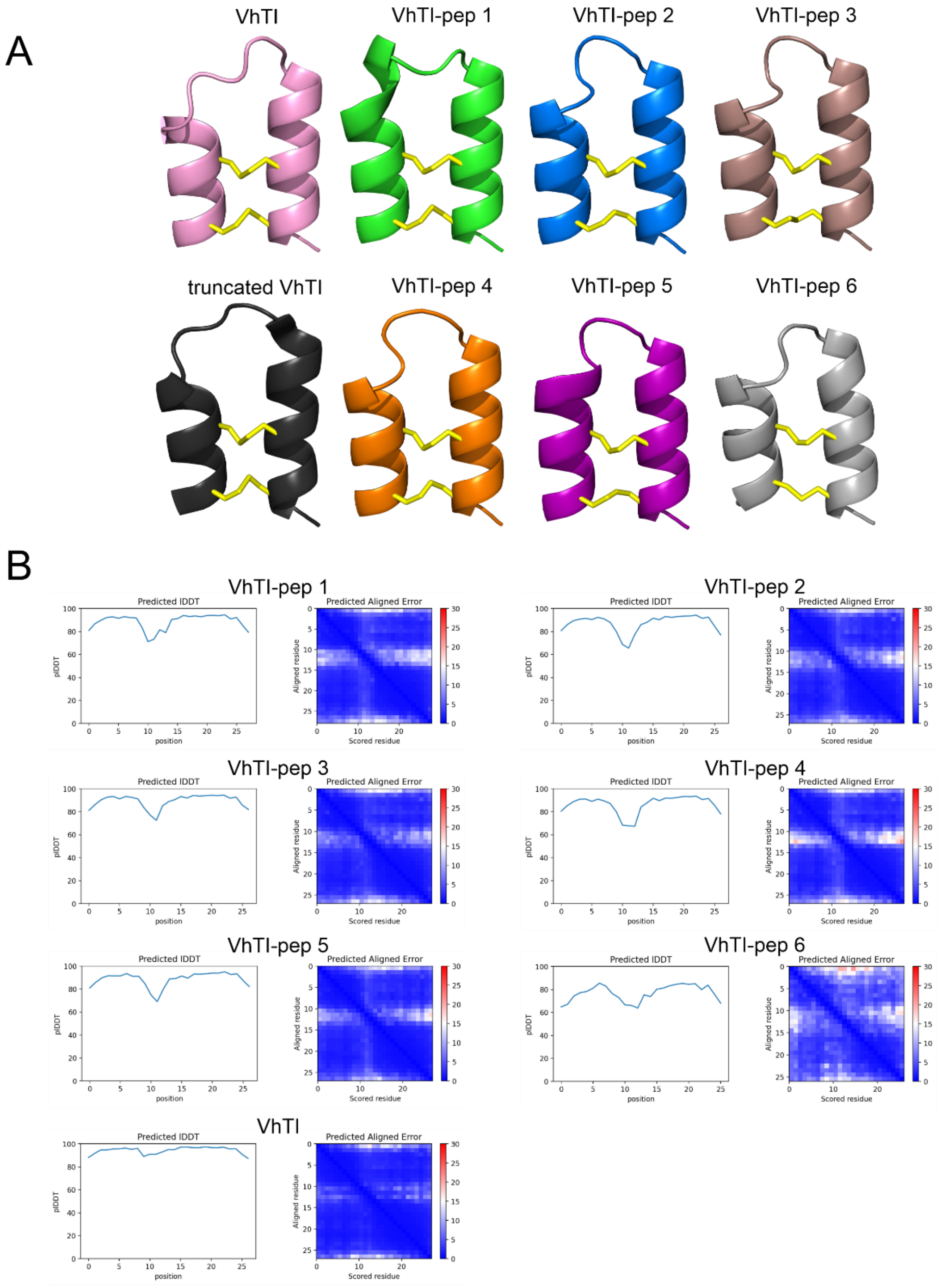

Figure S8. Serum stability and cytotoxicity of the peptide probes. **(A)** Serum stability of the linear pepitem, the VhTI variant as well as grafted VhTI-pep 1-6 was measured over 48 h. The stability was calculated as the % remaining peptide as compared to the native molecule (time point 0) as described in the methods section. Data are shown as mean ± SD. **(B)** Cytotoxicity of the peptides was tested at a concentration of 1 µM on HMEC-1 cells using CCK8 reagent, and absorbance at 450 nm was measured. The cytotoxic alkaloid camptothecin (150 µg/mL) was used as a positive control. Data are shown as mean ± SD for three independent measurements; statistical analysis was performed using one-way ANOVA with Dunnett's post hoc test. Statistical significance is indicated in graphs as: n.s. (not significant); *** *p*<0.001.

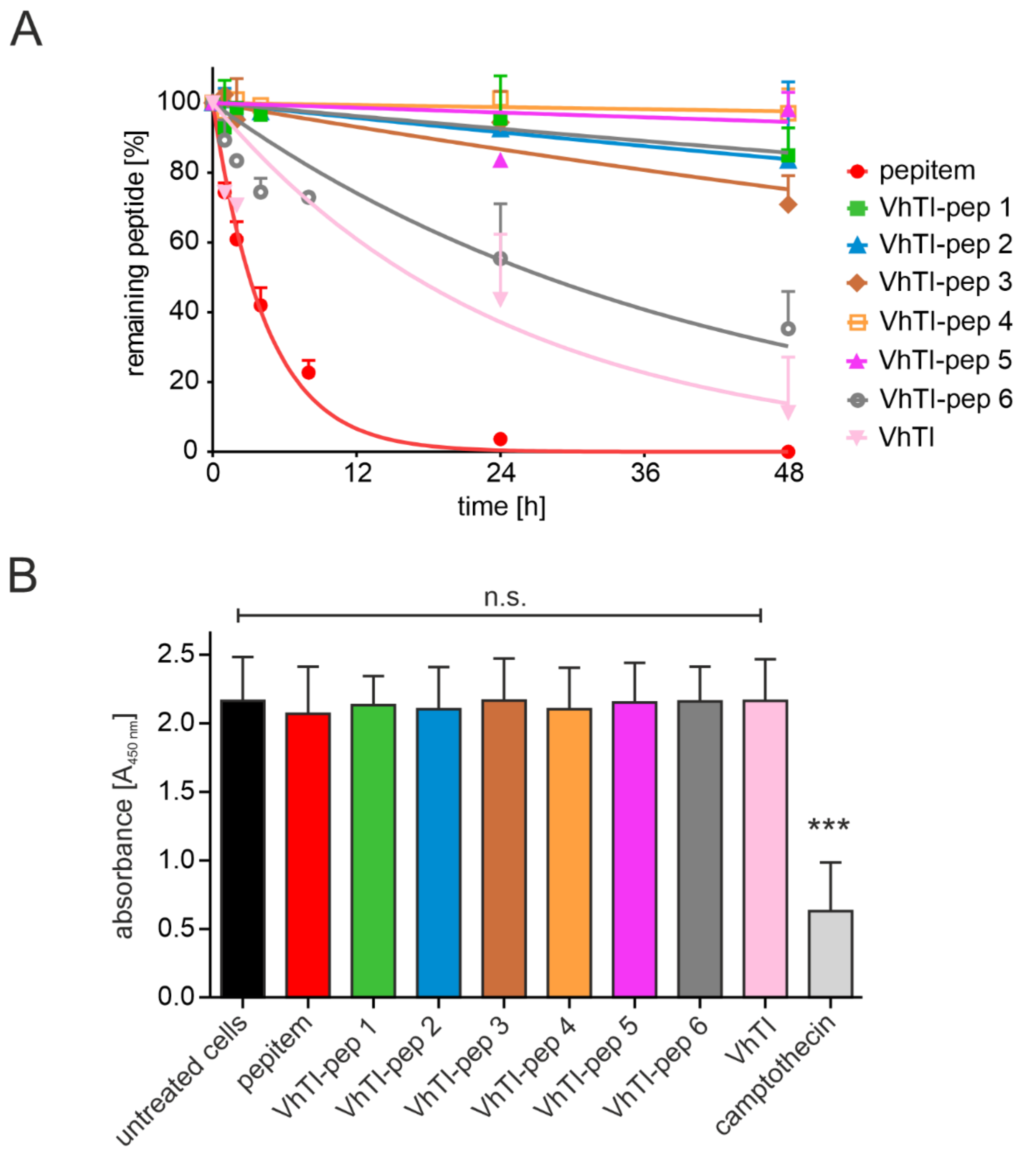

Figure S9. Structural analysis of VhTI-pep 2 using NMR. (A) The amide region of the TOCSY spectrum highlights the individual amino acid spin systems. The residue number is shown at the bottom of each spin system. **(B)** Amide region of the NOESY spectrum showing the 'sequential walk' of intra- and inter-residual Hα-HN cross peaks used for resonance assignment. Cross peaks are labelled next to the intra-residual peak. **(C)** ^{15}N HSQC spectrum. Correlations between amide protons and their corresponding amide nitrogens are labeled with their corresponding residue numbers. **(D)** ^{13}C HSQC spectrum highlighting ^{1}H-^{13}C correlations in side chain methylene groups. **(E)** Structural ensemble of the 20 best conformations. Side chains of the grafted amino acids (QGAEL) are shown in stick representation and with color-coding: Q (blue), G (red), A (magenta), E (green), and L (orange). **(F)** Random coil index graph showing the random coil index for the peptide residues, indicating amino acids 10-12 to be the least ordered residues in the peptide.

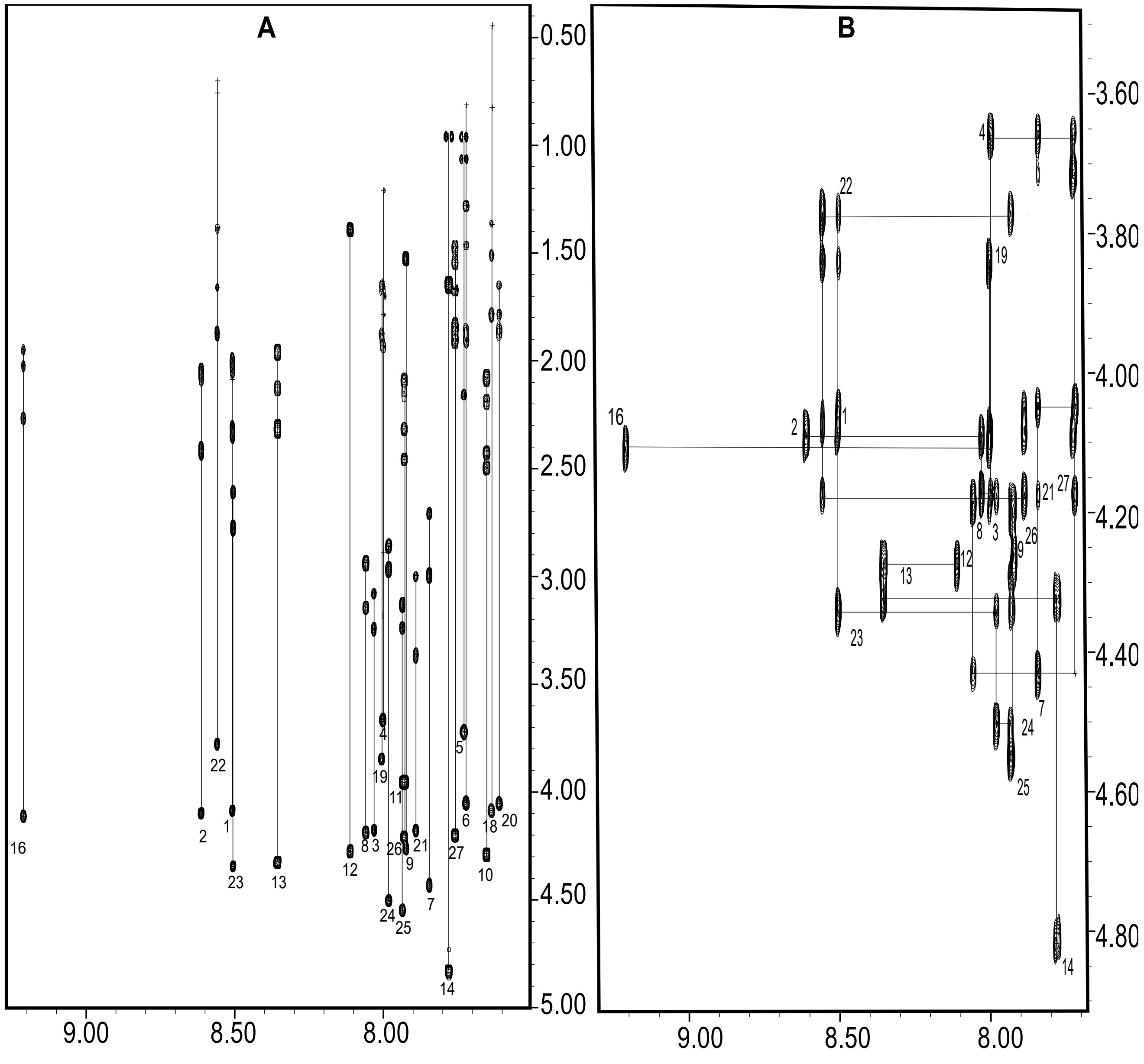

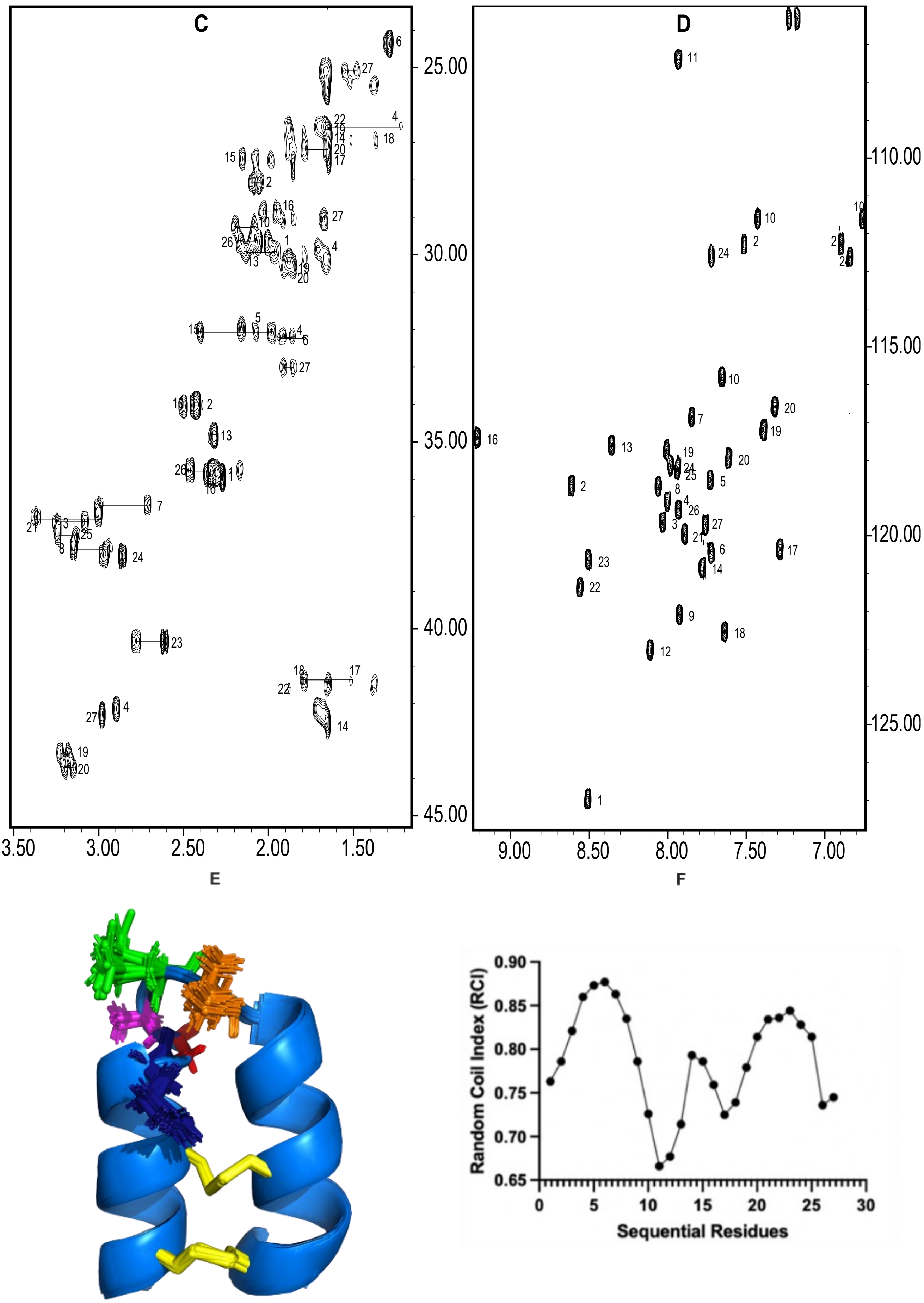
C
D
25.00
30.00
35.00
40.00
45.00
110.00
115.00
120.00
125.00
3.50
3.00
2.50
2.00
1.50
9.00
8.50
8.00
7.50
7.00
E
F
Random Coil Index (RCI)
0.90
0.85
0.80
0.75
0.70
0.65
0
5
10
15
20
25
30
Sequential Residues

Figure S10. EC$_{50}$ determination of VhTI-pep 2 for different T-cell subsets. **(A)** The peptide inhibited the migration of CD4$^+$ and CD8$^+$ lymphocytes **(B)** as well as their memory phenotypes. The cells were gated as CD3$^+$ and CD4$^+$/CD8$^+$, and for memory phenotypes as CD3$^+$, CD4$^+$/CD8$^+$, and CD45RO$^+$**.** Data are shown as mean ± SD for 4 independent measurements.

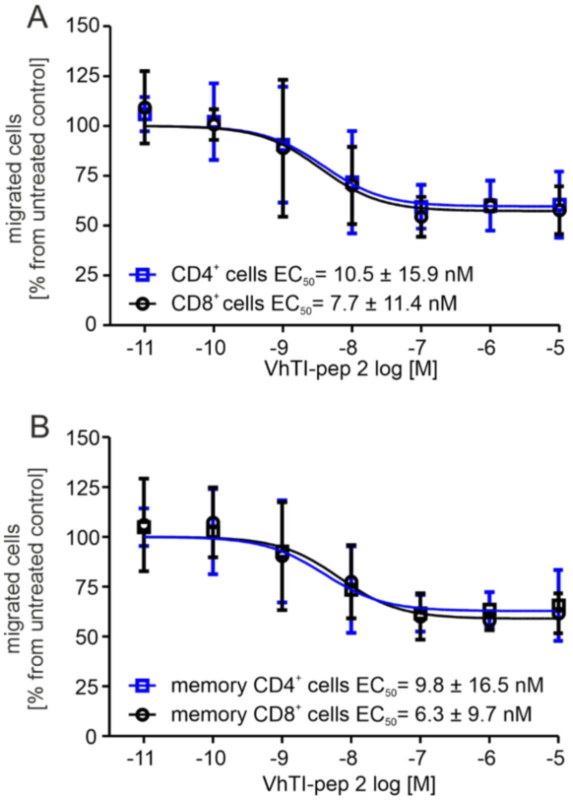

Figure S11. Peptide-protein interaction models between CDH15 and pepitem. Interactions between pepitem and **(A)** NCAM1 and **(B)** CDH15 were modeled using AF3. The predicted aligned error (PAE) matrix for the X-Y complex highlights regions of higher structural uncertainty. Local interaction area visualization shows regions of high local interaction score (LIS) within the complex. The 3D structure of the X-Y complex is color-coded by plDDT to indicate local structural confidence. The arrows indicate the position of the pepitem peptide.

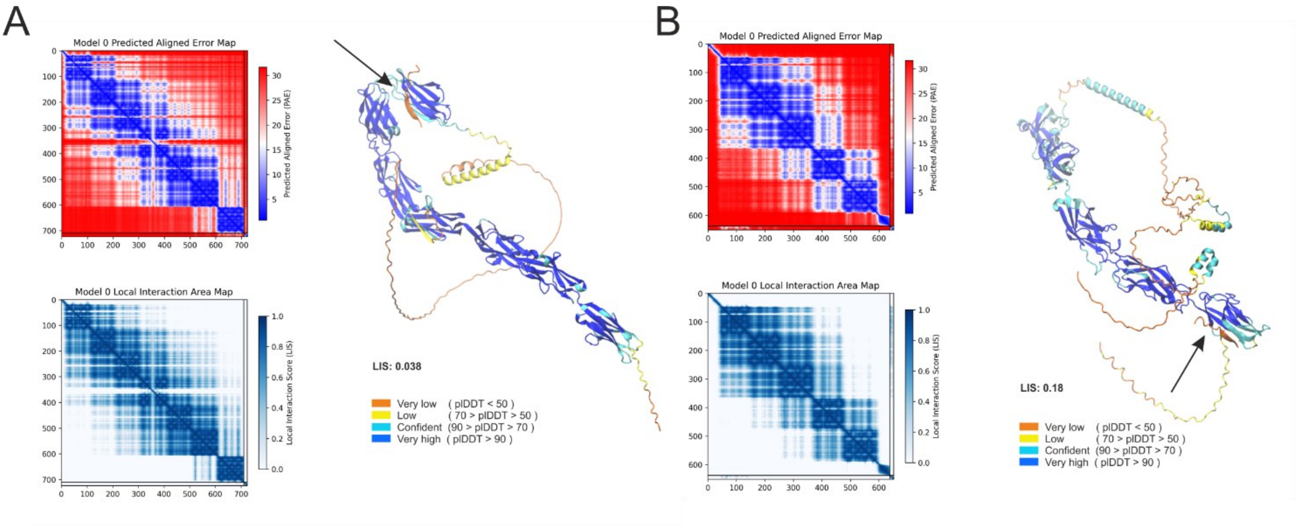

Figure S12. Peptide-protein interaction models between CDH15 and VhTI-pep 2. (A) Interaction of VhTI-pep 2 with the full-length CDH15 protein and **(B)** with the N-terminal CDH15 region. The five extracellular protein domains of CDH15 are labeled as ECD1-5. For all models, the predicted aligned error (PAE) matrix for the X-Y complex is shown, which highlights regions of higher structural uncertainty. The local interaction area visualization shows regions of high local interaction score (LIS) within the complex. The 3D structure of the X-Y complex is color-coded by plDDT to indicate local structural confidence. The arrows indicate the position of the peptide in the model. **(C)** Zoom-in on the ECD1-VhTI-pep 2 interface, showing the ECD1 of the CDH15 protein in green and the VhTI-pep 2 peptide in blue. **(D)** Two polar contacts forming between VhTI-pep 2 and CDH15 were predicted using PyMOL and are shown as yellow dashed lines, using the AF3 model generated for the interaction of the fragment ECD1 and the peptide. All amino acids involved in the interactions are labelled in one-letter code with their respective position in the peptide or CDH15 protein (the full-length protein was used as the reference for numbering the protein residues).

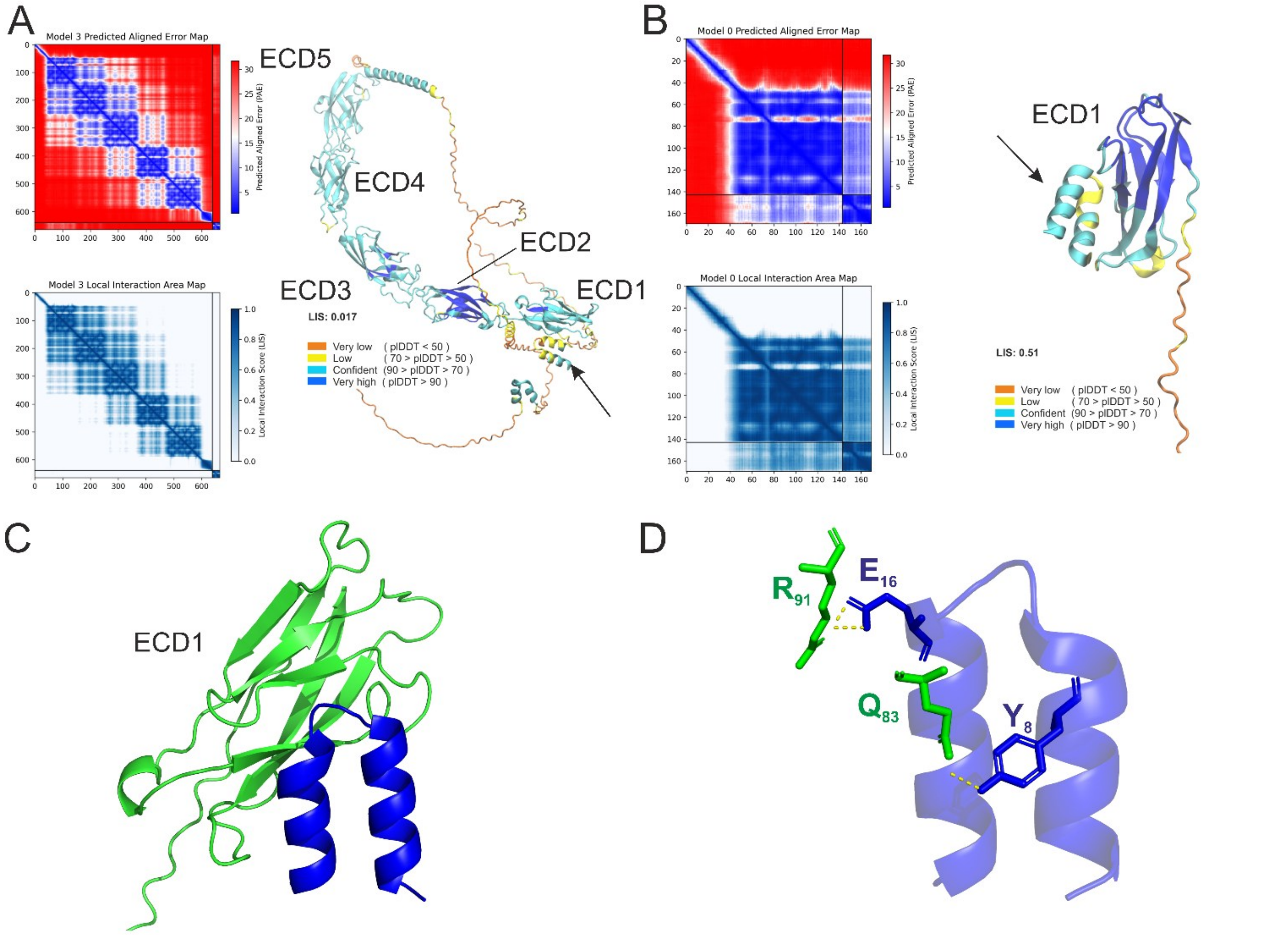

Figure S13. Western blot membranes. **(A-C)** Uncropped images of the Western blot membranes are shown, which were used to quantify VCAM-1 expression using β-actin as the loading control.

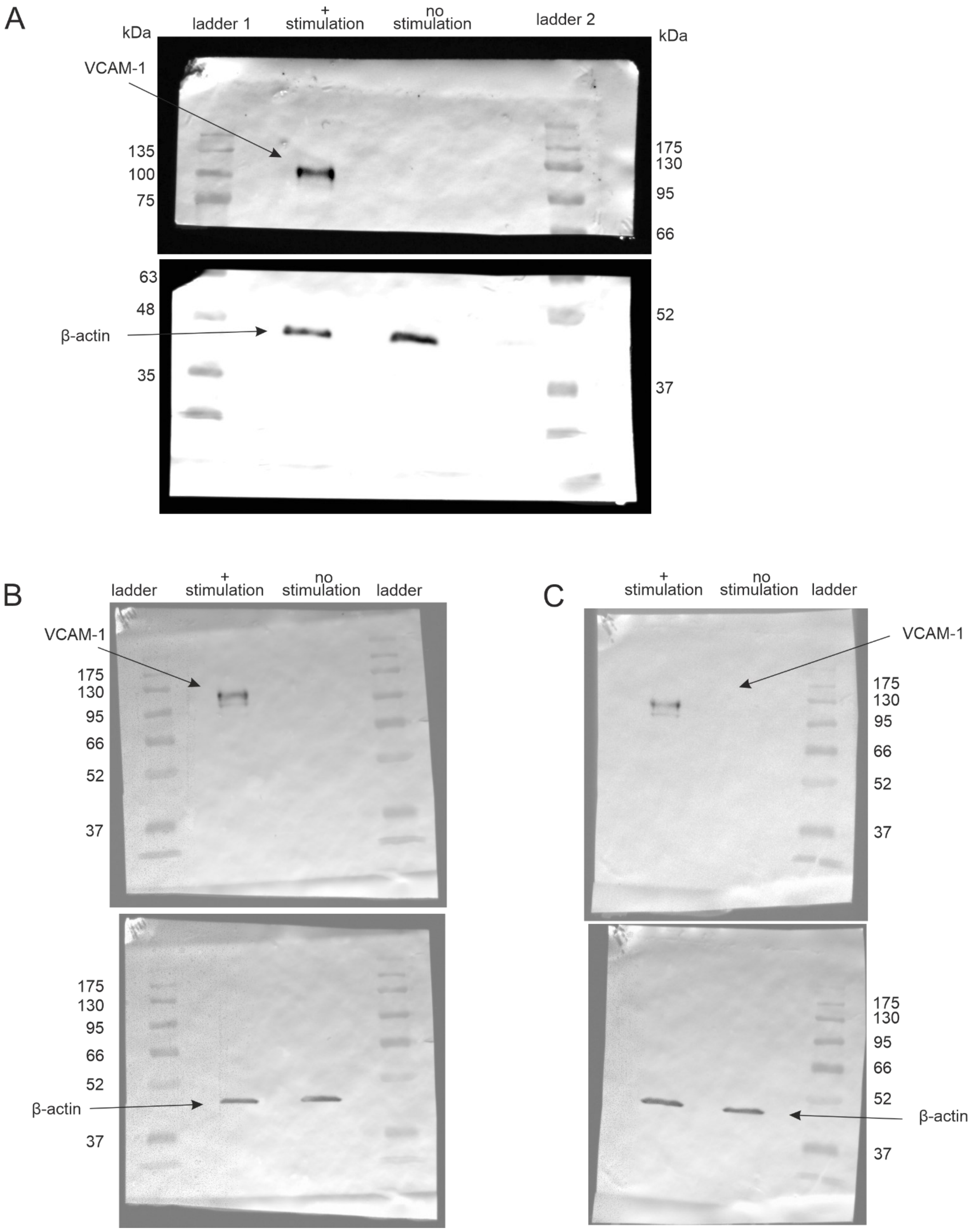

Table S1. Primer sequences used for quantitative RT-PCR.

Gene	Forward primer 5'-3'	Reverse primer 5'-3'	Reference
**VCAM-1**	CCGGATTGCTGCTCAGATTGGA	AGCGTGGAATTGGTCCCCTCA	[5]
**ICAM-1**	GCAGACAGTGACCATCTACAGCTT	CTTCTGAGACCTCTGGCTTCGT	[6]
**SphK1**	AATGAAGACCTCCTGACCAACTG	GACGCCGATACTTCTCACTCTCT	[7]
**CDH15**	GTCATCTACAGCATCCAGGG	AGGAAGGCTGGCCGGTTGTC	[8]
**Spns2**	ACTTTGGGGTCAAGGACCGA	AATCACCTTCCTGTTGAAGCG	[9]
**AktB**	AGAAAATCTGGCACCACACC	TAGCACAGCCTGGATAGCAA	[10]

Table S2. Pepitem sequence in chordata species.

See document in Supplementary information 2.

Table S3. Sequences of truncated pepitem probes and quality control of the synthesized peptides.

Peptide	Sequence	Peptide purity*	RT	Calc. $[M+H]^+$	Obs. $[M+H]^+$
**truncated pep-1**	SVTEQG	>95%	4.7 min	620.6	620.3
**truncated pep-2**	QGAELS	>91%[#]	11.3 min	604.3	604.3
**truncated pep-3**	LSNEER	>95%	7.2 min	747.4	747.4

* determined by analytical RP-HPLC analysis, monitoring absorbance at 214 nm. RT retention time on analytical RP-HPLC

[#]The purity could not be enriched further after two consecutive polishing HPLC purification runs.

Table S4. Identified hit peptides in the PDB structure similarity search.

See document in Supplementary information 2.

Table S5. RMSD calculation for alignment of the pepitem structure to scaffold peptides.

Peptide	PDB	RMSD (Å)#	Match align score	Atoms aligned	RMSD (Å)#	Match align score	Atoms aligned	RMSD (Å)#	Sequence
		global alignment*			helical framework alignment*				
		"super command"			"super command"			"cealign command"	
**VhTI**	2CMY	3.25	58.7	112	2.989	76.7	103	2.55	EQ**CKVMCYAQ**RHS**SPELLRRCLDNC**EKEHD
**BRSV-peptide$**	1BRV	0.33	28.6	31	3.367	31.2	32	n.d.	VP**CSTC**EGN**LACLSL**CHIE
**BWI-2c**	2LQX	4.87	99.0	132	4.411	87.6	109	4.65	SEKP**QQELEECQNVCRM**KRW**STEMVHRCEKKCEEK**FERQQR
**EcAMP1**	2L2R	3.96	50.5	117	0.375	19.6	45	3.25	GSGRG**SCRSQCMRRH**ED**EPWRVQECVSQCRR**RRGGGD
**Luffin P1**	2L37	4.36	94.4	135	3.565	75.8	116	2.71	GS**PRTEYEACRVRCQVAE**HG**VERQRRCQQVCEKRLREREG**RRE
**Tk-AMP-X2**	2M6A	3.92	49.3	114	3.149	59.6	79	n.d.	ADD**RCERM**CQRYHD**RREKKQCMKG**CRYG
**Nigellin-1.1**	2NB2	4.67	67.3	124	3.857	71.7	109	2.78	**DRYQDCLSECNSRCT**YIP**DYAGMRACIGLCAPACLTSR**
**truncated 14-3-3ζ**	1QJB								**AACMKSVTEQ**GAEL**SNEERNLLSV**

Peptides were aligned as indicated in the table either using the "super" or "cealign" command in PyMOL**.** * Alignments were performed either globally over the whole sequence, or the helical framework of the structures was selected and the alignment only performed over the helical part of the peptide structure. Helical residues are indicated in bold in the sequence. Residues not resolved in the structures are indicated in grey. Alignment was performed to a truncated 14-3-3ζ protein sequence as indicated with the pepitem amino acid sequence in red. $ Bovine respiratory syncytial virus-derived peptide. Alignments with peptide sequences, which were too short for the cealign command are indicated as n.d.

Table S6. Sequences of additionally modeled peptides.

Peptide	Sequence
**VhTI-pep 7**	EQCKVMC----**EQGA-LS**ELLRRC------LDNCEK
**VhTI-pep 8**	EQCKVMC**SVT-EQGAELS**-------**NEER**CLDNCEK
**VhTI-pep 9**	EQ----C**SVT**C**EQGAEL**PELLRRC**SNEER**----CEK

Table S7. Alignment of predicted VhTI and VhTI-pep 1-6 structures.

Peptide 1	Peptide 2	RMSD (Å)
**VhTI (PDB: 2PLX)*,#**	VhTI (AlphaFold)	0.51
	VhTI-pep 1	0.43
	VhTI-pep 2	0.59
	VhTI-pep 3	0.59
	VhTI-pep 4	0.50
	VhTI-pep 5	0.64
	VhTI-pep 6	1.25
	VhTI-pep 7	0.76
	VhTI-pep 8	1.88
	VhTI-pep 9$	n.d.

*For prediction of the peptide structures using AlphaFold2, the norleucine of the grafted peptides and the VhTI variant were substituted with a methionine, acetylation and amidation of the N- and C-terminus, respectively, were not taken into account. The RMSD values were calculated using the "align" command in PyMOL. #The VhTI model derived from the PDB: 2PLX deposit data lacks the Glu residue on position 1. $The design did not provide secondary structure using AlphaFold and was therefore not used for alignment.

Table S8. NMR structural statistics for VhTI-Pep2.

	VhTI-Pep2
**Distance restraints**	
Intra residual (|i-j| = 0)	235
Sequential (|i-j| = 1)	161
Medium range (1 < |i-j| < 5)	219
Long range (|i-j| ≥ 5)	84
Hydrogen bonds	28
Total	727
**Dihedral angle restraints**	
Φ	23
Ψ	23
Χ	15
Total	61
**Energies (kcal/mol, mean ± SD)**	
Overall	-1154.1 ± 28.9
Bonds	9.47 ± 0.96
Angles	46.39 ± 3.95
Improper	14.03 ± 2.22
Dihedral	132.7 ± 1.51
Van der Waals	-121.9 ± 5.09
Electrostatic	-1235.1 ± 32.35
NOE (experimental)	0.125 ± 0.022
Constrained dihedrals (experimental)	0.197 ± 0.172
**Atomic RMSD (Å)**	
Mean global backbone (6-30,41-52)	0.31 ± 0.07
Mean global heavy atoms (6-30,41-52)	0.99 ± 0.12
**MolProbity**	
Clash Score, all atoms	19.08 ± 6.50
Poor rotamers (%)	0.46 ± 1.40
Favored rotamers (%)	88.64 ± 5.99
Ramachandran Outliers (%)	0.00 ± 0.00
Ramachandran Favored (%)	100.00 ± 0.00
MolProbity score	1.81 ± 0.18
MolProbity score percentile	84.10 ± 8.05
**Violations from experimental restraints**	
NOE violations exceeding 0.2 Å	0
Dihedral violations exceeding 2°	0

Table S9. Proposed protein-protein interactions between NCAM-1 and VhTI-pep 2 using the protein-ligand interaction profiler online tool.

Hydrophobic interactions					
**Index**	**Residue**	**AA**	**Distance (Å)**	**Ligand Atom**	**Protein Atom**
**1**	387A	TYR	3.52	4754	1274
**2**	411A	GLU	3.66	4658	1613
**3**	439A	PHE	3.72	4849	2042
**4**	441A	TYR	3.68	4658	2077
**5**	441A	TYR	3.82	4702	2074
**6**	469A	PRO	3.58	4925	2519

Hydrogen bonds									
**Index**	**Residue**	**AA**	**Distance H-A (Å)**	**Distance A-H (Å)**	**Donor angle**	**Protein donor**	**Side chain**	**Donor atom**	**Acceptor atom**
**1**	387A	TYR	2.77	3.26	112.47	Yes	Yes	1276 [O3]	4768 [O.co2]
**2**	441A	TYR	2.11	3.02	148.93	No	Yes	4586 [N3]	2079 [O3]
**3**	443A	SER	2.17	3.09	158	No	Yes	4706 [O2]	2108 [O3]

Interactions were proposed using the protein-ligand interaction profiler online tool [11] using the AF3-generated fragment of Ncam-1, consisting of Ig4, Ig5, and FN1, and VhTI-pep2 as input (see Figure 6B). Residue numbers are derived from the full-length Ncam-1 protein.

Table S10. AlphaFold-Multimer prediction of interacting residues between VhTI-pep 2 and target proteins.

Binding protein#	Protein residue	Peptide residue	Distance (Å)	pIDDT score range
NCAM1	TYR 387	GLU 16	3.22	< 50
	LYS 417	LEU 14	3.12	< 50
	GLN 419	LEU 17	2.30	50-70
	GLN 419	CYS 21	3.15	50-70
	GLY 420	GLN 10	3.26	< 50
	PRO 421	GLN 10	2.53	< 50
	VAL 422	GLN 10	3.35	< 50
	GLU 437	ASN 24	2.69	50-70
	PHE 439	LEU 17	2.01	< 50
	PHE 439	ARG 20	3.23	50-70
NCAM1 fragment	TYR 388	ALA 12	3.03	50-70
	ALA 391	ALA 9	2.82	50-70
	GLU 393	MET 6	3.28	50-70
	TYR 410	GLN 2	2.97	50-70
	LEU 411	VAL 5	3.21	50-70
	GLU 412	VAL 5	3.34	50-70
	TYR 415	TYR 8	3.06	50-70
	ALA 441	TYR 8	2.71	70-90
	TYR 442	GLU 1	2.94	50-70
	SER 471	PRO 15	3.34	50-70
	SER 471	ARG 19	3.27	70-90
CDH15	TRP 47	GLN 2	3.02	50-70
	TYR 63	ALA 12	3.20	< 50
	GLN 67	VAL 5	3.25	50-70
CDH15 fragment	GLN 83	TYR 8	2.30	70-90
	GLN 83	LEU 22	3.29	50-70
	VAL 87	PRO 15	2.69	70-90
	ARG 92	GLU 16	2.33	50-70
	ARG 92	ARG 19	3.00	50-70
	ILE 97	GLU 13	3.18	50-70
	LEU 125	TYR 8	3.35	70-90
	GLY 129	VAL 5	3.05	50-70

The binding proteins refer to the different models using either the full protein or specific fragment analysis, as seen in Figure 6.

2 Additional References

[1] J. Jaczewska, M.H. Abdulreda, C.Y. Yau, M.M. Schmitt, I. Schubert, P.-O. Berggren, et al., TNF-α and IFN-γ promote lymphocyte adhesion to endothelial junctional regions facilitating transendothelial migration. J. Leukoc. Biol. 95(2) 2014; pp. 265-274. https://doi.org/10.1189/jlb.0412205

[2] C.J. Murphy, E. Bentley, P.E. Miller, K. McIntyre, G. Leatherberry, R. Dubielzig, et al., The Pharmacologic Assessment of A Novel Lymphocyte Function-Associated Antigen-1 Antagonist (SAR 1118) for the Treatment of Keratoconjunctivitis Sicca in Dogs. Investigative Ophthalmology & Visual Science 52(6) 2011; pp. 3174-3180. https://doi.org/10.1167/iovs.09-5078

[3] J. Jumper, R. Evans, A. Pritzel, T. Green, M. Figurnov, O. Ronneberger, et al., Highly accurate protein structure prediction with AlphaFold. Nature 596(7873) 2021; pp. 583-589. https://doi.org/10.1038/s41586-021-03819-2

[4] S.A. Rettie, K.V. Campbell, A.K. Bera, A. Kang, S. Kozlov, Y.F. Bueso, et al., Cyclic peptide structure prediction and design using AlphaFold2. Nat. Commun. 16(1) 2025; p. 4730. 10.1038/s41467-025-59940-7

[5] S. Kotla, H.T. Vu, K.A. Ko, Y. Wang, M. Imanishi, K.-S. Heo, et al., Endothelial senescence is induced by phosphorylation and nuclear export of telomeric repeat binding factor 2–interacting protein. JCI Insight 4(9) 2019. https://doi.org/10.1172/jci.insight.124867

[6] J. Tu, Z. Hu, Z. Chen, Endothelial Gene Expression and Molecular Changes in Response to Radiosurgery in In Vitro and In Vivo Models of Cerebral Arteriovenous Malformations, Biomed Res. Int. 2013(1) 2013; 408253. https://doi.org/10.1155/2013/408253

[7] J. Yang, C. Yang, S. Zhang, Z. Mei, M. Shi, S. Sun, et al., ABC294640, a sphingosine kinase 2 inhibitor, enhances the antitumor effects of TRAIL in non-small cell lung cancer. Cancer Biol. Ther. 16(8) 2015; pp. 1194-1204. https://doi.org/10.1080/15384047.2015.1056944

[8] S. Charrasse, F. Comunale, E. Gilbert, O. Delattre, C. Gauthier-Rouvière, Variation in cadherins and catenins expression is linked to both proliferation and transformation of Rhabdomyosarcoma. Oncogene 23(13) 2004; pp. 2420-2430. https://doi.org/10.1038/sj.onc.1207382

[9] S. Fukuhara, S. Simmons, S. Kawamura, A. Inoue, Y. Orba, T. Tokudome, et al., The sphingosine-1-phosphate transporter Spns2 expressed on endothelial cells regulates lymphocyte trafficking in mice. J. Clin. Investig. 122(4) 2012; pp. 1416-1426. https://doi.org/10.1172/JCI60746

[10] T. Li, H. Diao, L. Zhao, Y. Xing, J. Zhang, N. Liu, et al., Identification of suitable reference genes for real-time quantitative PCR analysis of hydrogen peroxide-treated human umbilical vein endothelial cells. BMC Mol. Biol. 18(1) 2017; p. 10. https://doi.org/10.1186/s12867-017-0086-z

[11] M.F. Adasme, K.L. Linnemann, S.N. Bolz, F. Kaiser, S. Salentin, V J. Haupt, et al., PLIP 2021: expanding the scope of the protein–ligand interaction profiler to DNA and RNA. Nucleic Acids Res. 49(W1) 2021; pp. W530-W534. http://dx.doi.org/10.1093/nar/gkab294
